# Identification of a Conserved Transcriptional Activator-Repressor Module Controlling the Expression of Genes Involved in Tannic Acid Degradation and Gallic Acid Utilization in *Aspergillus niger*

**DOI:** 10.3389/ffunb.2021.681631

**Published:** 2021-05-25

**Authors:** Mark Arentshorst, Marcos Di Falco, Marie-Claude Moisan, Ian D. Reid, Tessa O. M. Spaapen, Jisca van Dam, Ebru Demirci, Justin Powlowski, Peter J. Punt, Adrian Tsang, Arthur F. J. Ram

**Affiliations:** ^1^Molecular Microbiology and Biotechnology, Institute of Biology Leiden, Leiden University, Leiden, Netherlands; ^2^Centre for Structural and Functional Genomics, Concordia University, Montreal, QC, Canada; ^3^Department of Chemistry & Biochemistry, Concordia University, Montreal, QC, Canada; ^4^Dutch DNA Biotech, Hugo R Kruytgebouw 4-Noord, Utrecht, Netherlands

**Keywords:** ring cleavage enzyme, tannases, monooxygenase, Zn(II)_2_Cys_6_ transcriptional activator, gene regulation, ring cleaving enzymes, tannases, monooxygenase

## Abstract

Tannic acid, a hydrolysable gallotannin present in plant tissues, consists of a central glucose molecule esterified with gallic acid molecules. Some microorganisms, including several *Aspergillus* species, can metabolize tannic acid by releasing gallic acid residues from tannic acid by secreting tannic acid specific esterases into the medium. The expression of these so-called tannases is induced by tannic acid or gallic acid. In this study, we identified a conserved transcriptional activator-repressor module involved in the regulation of predicted tannases and other genes involved in gallic acid metabolism. The transcriptional activator-repressor module regulating tannic acid utilization resembles the transcriptional activator-repressor modules regulating galacturonic acid and quinic acid utilization. Like these modules, the Zn(II)_2_Cys_6_ transcriptional activator (TanR) and the putative repressor (TanX) are located adjacent to each other. Deletion of the transcriptional activator *(*Δ*tanR*) results in inability to grow on gallic acid and severely reduces growth on tannic acid. Deletion of the putative repressor gene *(*Δ*tanX*) results in the constitutive expression of tannases as well as other genes with mostly unknown function. Known microbial catabolic pathways for gallic acid utilization involve so-called ring cleavage enzymes, and two of these ring cleavage enzymes show increased expression in the Δ*tanX* mutant. However, deletion of these two genes, and even deletion of all 17 genes encoding potential ring cleavage enzymes, did not result in a gallic acid non-utilizing phenotype. Therefore, in *A. niger* gallic acid utilization involves a hitherto unknown pathway. Transcriptome analysis of the Δ*tanX* mutant identified several genes and gene clusters that were significantly induced compared to the parental strain. The involvement of a selection of these genes and gene clusters in gallic acid utilization was examined by constructing gene deletion mutants and testing their ability to grow on gallic acid. Only the deletion of a gene encoding an FAD-dependent monooxygenase (NRRL3_04659) resulted in a strain that was unable to grow on gallic acid. Metabolomic studies showed accumulation of gallic acid in the Δ*NRRL3_04659* mutant suggesting that this predicted monooxygenase is involved in the first step of gallic acid metabolism and is likely responsible for oxidation of the aromatic ring.

## Introduction

Tannins are polyphenolic aromatic compounds present in plant tissues such as leaves, bark, and wood (Aguilar et al., 2007). In plants, tannins are able to form strong complexes with proteins and polysaccharides like starch and cellulose to protect plants from degradation by fungal enzymes (Lekha and Lonsane, 1997). Tannic acid is a hydrolysable gallotannin, and consists of a central glucose molecule esterified with on average eight gallic acid molecules (Chávez-González et al., 2012).

Some microorganisms can use tannic acid as a carbon source. To utilize tannic acid, these microorganisms secrete tannin acyl hydrolases (also called tannases). Tannases catalyze the hydrolysis of the carboxylic ester bonds, resulting in the degradation of tannic acid to release gallic acid. Microorganisms that are capable of producing tannases include the bacterium *Pseudomonas putida* (Nogales et al., 2011), the yeast *Blastobotrys (Arxula) adeninivorans* (Meier et al., 2017) as well as several filamentous fungi of which *Aspergillus* species have been studied the most (Pinto et al., 2001; van Diepeningen et al., 2004). Although the amino acid sequences of bacterial, yeast, and fungal tannases are rather divergent, they all contain the active site motif of Gly-X-Ser-X-Gly, which is a characteristic of serine hydrolases (de las Rivas et al., 2019). The expression of fungal tannases is transcriptionally controlled. Their expression is induced when tannins or some of their hydrolysis products–such as gallic acid, pyrogallol, or methyl gallate–are present (Bajpai and Patil, 1997; Lekha and Lonsane, 1997; Aguilar et al., 2007). However, gallic acid was also reported to repress tannase synthesis under solid state fermentation conditions in *Aspergillus niger* (Aguilar et al., 2001).

Based on available data, mainly derived from studies in bacteria, the ring structure in aromatic compounds, including gallic acid, is considered to be opened by specific so-called ring cleavage enzymes. Up to now, seven major monocyclic aromatics have been shown to be substrates of specific ring cleavage enzymes [reviewed by Lubbers et al. (2019b)]. These substrates, besides gallic acid, include catechol, protocatechuic acid, hydroxyquinol, hydroquinone, gentisic acid, and pyrogallol. After oxygenation, the ring-opened compounds are further metabolized and channeled into central metabolism (Lubbers et al., 2019b).

Two mechanisms of ring cleavage have been described and are catalyzed by either intradiol or extradiol ring cleavage dioxygenases. Ring cleavage using the intradiol dioxygenases, also called *ortho*-cleavage dioxygenases, takes place between hydroxyl groups of the ring structure, while the extradiol- or *meta-*cleavage dioxygenases cleave adjacent to a hydroxyl group (Harwood and Parales, 1996). Bacterial species use both intra- and extradiol dioxygenases for the catabolism of aromatic compounds, but in fungi only intradiol cleavage has been described (e.g., Cain et al., 1968). In *A. niger*, four aromatic intradiol ring cleavage dioxygenases have been characterized for the catabolism of hydroxyquinol, catechol, and protocatechuate (Semana and Powlowski, 2019). The involvement of certain ring opening enzymes involved in protocatechuate was further confirmed by deletion analysis (Lubbers et al., 2019c). In the research presented here, we have identified several additional genes annotated as putative extradiol dioxygenase in the genome of *A. niger* whose encoding enzymes have not yet been studied.

**Figure 1:**
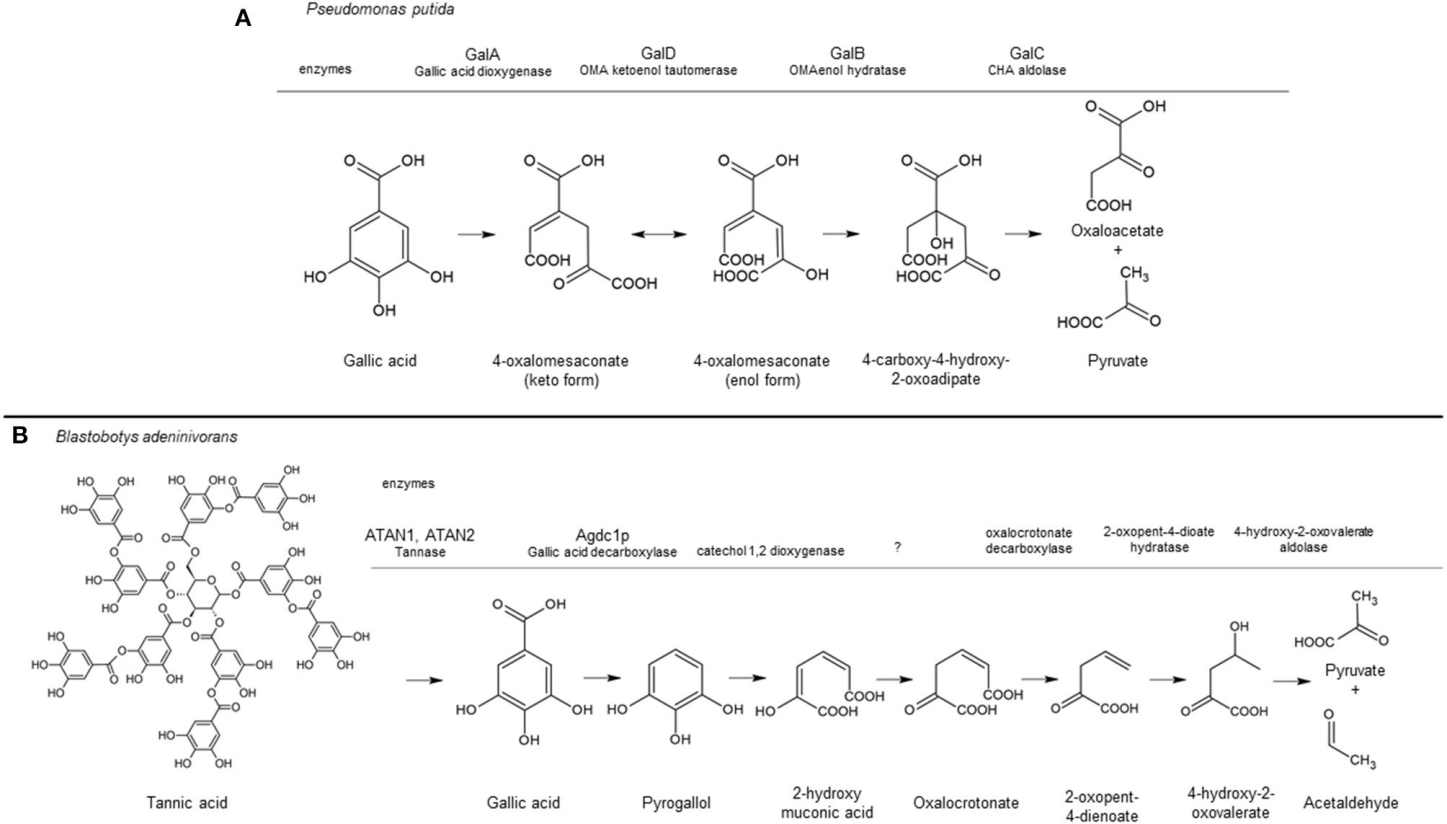
Tannic acid/gallic acid catabolic pathway in *Pseudomonas putida* **(A)** and *Blastobotys adeninivorans* **(B)** based on Nogales et al. (2011) and Meier et al. (2017), respectively. The aromatic converting enzymes are indicated above the gray line corresponding to their conversion step in the pathway, including the enzyme name when identified. A question mark indicates the enzyme has not being identified. This figure was made using ACD/ChemSketch Freeware, version 2019.2.1, Advanced Chemistry Development, Inc., Toronto, ON, Canada, www.acdlabs.com, 2019.

The catabolic pathway of gallic acid in bacteria and yeast species has been studied in detail and shown in Figure 1. In *P. putida*, ring cleavage of gallic acid is catalyzed by the Fe^2+^-dependent extradiol dioxygenase GalA, resulting in the intermediate 4-oxalomesaconic acid (OMA)keto, which is converted into OMAenol by the GalD isomerase. After hydration of OMAenol by the Zn^2+^-dependent hydratase GalB, the degradation of the intermediate 4-carboxy-4-hydroxy-2-oxoadipic acid is catalyzed by GalC into pyruvic acid and oxaloacetic acid which channel into central metabolism (Nogales et al., 2011). A different metabolic pathway has been identified for the yeast *B. adeninivorans*. In this pathway, gallic acid is decarboxylated to form pyrogallol by the gallic acid decarboxylase Agdc1p. Ring cleavage of pyrogallol is believed to be catalyzed by a still unidentified catechol 1,2 dioxygenase to produce 2-hydroxymuconic acid, which is further degraded into pyruvate and acetaldehyde (Meier et al., 2017). Apart from Agdc1p, no other genes or gene products involved in the catabolism of gallic acid in *B. adeninivorans* have been identified.

Knowledge about the degradation pathways of aromatic acids in filamentous fungi is limited. Best studied is the 3-oxoadipate pathway, or β-ketoadipate pathway, which has branches for the catabolism of protocatechuate and catechol using intradiol cleavage and channeling the products via β-ketoadipate into the TCA cycle (Martins et al., 2015; Lubbers et al., 2019c, 2021). As indicated above, the intradiol dioxygenases involved have been characterized by Semana and Powlowski (2019) and Lubbers et al. (2019c). Additional enzymes involved in catabolic steps after ring cleavage of protocatechuic and salicylic have been identified in *A. nidulans* by proteomic, transcriptomics and deletion analyses (Martins et al., 2015) and in *A. niger* (Lubbers et al., 2019c, 2021). The catabolic pathway for cinnamic acid in *A. niger* has also been studied. Three enzymes play a role in cinnamic acid and sorbic acid degradation, but are not required for the catabolism of other related aromatic compounds (Lubbers et al., 2019a). Genes involved in the metabolism of gallic acid in filamentous fungi have yet not been reported.

Biochemical studies on the catabolism of gallic acid have been performed in *A. niger* and *A. flavus* (Watanabe, 1965; Gurujeyalakshmi and Mahadevan, 1987). Catabolic pathways for gallic acid were proposed based on the identification of intermediates. For *A. niger*, it is proposed that gallic acid is oxidized to a peroxide and decomposed to cis-aconitic acid and trans-aconitic acid, which can be decarboxylated to α-ketoglutaric acid (Watanabe, 1965). In *A. flavus*, the intermediates 4-carboxy-2-hydroxy-cis, cis-muconic acid (4-oxalomesaconate) and pyruvic acid were detected during gallic acid degradation, which suggests the involvement of an extradiol gallate dioxygenase enzyme (Gurujeyalakshmi and Mahadevan, 1987).

Saprophytic fungi, including *A. niger*, produce an arsenal of extracellular enzymes to degrade plant derived biomass (Pel et al., 2007; de Vries et al., 2017). The enzymes involved in the utilization and catabolism of plant compounds by fungi is tightly controlled. Transcription factors activate the expression of target genes, involved in specific metabolic pathways, by binding to specific transcription factor binding sites located upstream of these genes (Benocci et al., 2017). With such a controlled regulation system, transcriptional activation of substrate specific enzymes, transporters and catabolic pathway enzymes is only activated when a particular plant compound is present. Well-studied examples in *A. niger* are the transcription factors AmyR and XlnR that control the expression of genes encoding enzymes involved in starch and xylan utilization, respectively (van Peij et al., 1998; Petersen et al., 1999; van Kuyk et al., 2012). Recently, the regulatory mechanism of genes involved in the utilization of pectin and its main component galacturonic acid was studied (Alazi et al., 2016, 2017; Niu et al., 2017). These studies indicate that the galacturonic acid catabolic pathway in *A. niger* is controlled by a Zn(II)_2_Cys_6_ transcriptional activator (GaaR) in combination with a specific repressor protein (GaaX) (Niu et al., 2017). The activator (GaaR) and the corresponding repressor (GaaX) are located next to each other in the genome and form a module controlling the expression of genes involved in galacturonic acid utilization. Under non-inducing conditions, GaaX is expected to repress GaaR activity, thereby blocking the expression of target genes involved in the utilization of pectin and catabolism of galacturonic acid. A similar activator-repressor module for the degradation of quinic acid consisting of the Zn(II)_2_Cys_6_ transcriptional activator QutA/QA-1F and the corresponding repressor QutR/QA-1S is found in *A. nidulans* and *Neurospora crassa*, respectively (Giles et al., 1985; Geever et al., 1989; Levesley et al., 1996). When quinic acid or quinic acid derived metabolite binds to the repressor, it is expected to cause allosteric change of the repressor protein, negating its repressing function and activating the expression of target genes via activating the quinic acid activator (Lamb et al., 1996). A similar quinic acid module is also present in *A. niger* (Niu et al., 2017).

In this study, key players in the regulatory mechanism related to tannic acid utilization and gallic acid catabolism have been identified in *A. niger*. Its transcriptional control is mediated by an activator TanR and the corresponding putative repressor TanX. Like the GaaR/GaaX and QutA/QutR activator-repressor modules, TanR [Zn(II)_2_Cys_6_ activator] and TanX (repressor) are located next to each other in the genome.

## Materials and Methods

### Strains, Media, and Growth Conditions

The *A. niger* strains used in this study are listed in Table 1. Strains were grown on liquid or solidified [containing 1.5 % (w/v) Scharlau agar] minimal medium (MM) or on complete medium (CM) as described (Arentshorst et al., 2012). *Aspergillus niger* transformants were purified as described (Arentshorst et al., 2012) using a final concentration of 100 μg/mL hygromycin. Mycelial growth assays were performed on MM plates, usually supplemented with 5 mM of the aromatic compound. Aromatic compounds were weighed, dissolved in sterile water and mixed with an equal volume of 2 x concentrated solidified MM. Radial growth of *A. niger* strains was assayed by point inoculation of 5 μL filtered spore suspension (2*10^5^ spores/mL) in the center of an agar plate and incubation of the plates for 5 days at 30°C. *Escherichia coli* DH5α was used for plasmid construction and cultured at 37°C in Luria-Bertani medium, with ampicillin (100 μg/mL).

**Table 1:** Strains used in this study.

**Strain**	**Genotype**	**References**
N402	*cspA*	Bos et al., 1988
MA169.4	*cspA, pyrG378, kusA::DR-amdS-DR*	Carvalho et al., 2010
MA234.1	*cspA, kusA::DR-amdS-DR*	Alazi et al., 2016
MA514.1	*tanR::hygR* in MA234.1	This study
MA586.1	*tanX::hygR* in MA234.1	This study
TS6.4	*NRRL3_04659::AopyrG* in MA169.4	This study
MA831.1	*NRRL3_06002::AopyrG* in MA169.4	This study
TS2.1	*NRRL3_08632::AopyrG* in MA169.4	This study
MA835.1	*NRRL3_04277::hygB* in TS2.1	This study
MA829.1	*NRRL3_01651-01656::AopyrG*	This study
MA852.1	*NRRL3_02180-02185::AopyrG*	This study
TS10.1	*NRRL3_06147-06150::AopyrG*	This study
MA857.2	*NRRL3_08290-08292::AopyrG*	This study
MA963.4	*NRRL3_09054-09057::AopyrG*	This study
MA860.2	*NRRL3_10365-10375::AopyrG*	This study
MA925.2	Δ*NRRL3_00414, ΔNRRL3_00984, ΔNRRL3_01405, ΔNRRL3_02496, ΔNRRL3_02522, ΔNRRL3_02644, ΔNRRL3_02896, ΔNRRL3_03141, ΔNRRL3_03148, ΔNRRL3_03326, ΔNRRL3_04277, ΔNRRL3_04335, ΔNRRL3_04787, ΔNRRL3_05330, ΔNRRL3_05564, ΔNRRL3_08632, ΔNRRL3_11743* in MA234.1	This study

### Bioreactor Cultures, RNA Isolation, and RNAseq

Bioreactor controlled batch fermentation on fructose of the Δ*tanX* strain MA586.1 was performed as described previously (Jørgensen et al., 2010; Niu et al., 2017). In short, autoclaved bioreactor vessels were filled with 5 L of sterile MM containing 0.75% fructose as a carbon source. During cultivation at 30°C, the controller was set to maintain pH 3 by addition of titrants (2 M NaOH or 1 M HCl). Sterile air was supplied at a rate of 1 L min^−1^. Prior to inoculation, 1.5 ml of 10% (w/v) filter-sterilized yeast extract was added to enhance conidial germination. Cultures were inoculated with freshly harvested spores at a concentration of 7.0 × 10^8^ conidia per liter. To reduce the loss of hydrophobic conidia during germination, the stirrer speed was set to 250 rpm and the culture was aerated via the headspace during the first 6 h after inoculation. Subsequently, the stirrer speed was increased to 750 rpm, 0.5 ml of polypropyleneglycol P2000 was added as an antifoam agent and air was supplied via the sparger. Cultures broth was harvested at regular intervals from batch cultures and mycelial biomass was retained by vacuum filtration using glass microfiber filters (Whatman). Both biomass and filtrate were quickly frozen in liquid nitrogen and subsequently stored at −80°C. Dry biomass concentrations were gravimetrically determined from lyophilized mycelia originating from a known mass of culture broth. RNA was isolated from mycelium that was grown until mid-exponential phase. Extraction of RNA, library construction and RNA sequencing were performed as described (Niu et al., 2017). Three biological replicates for the reference strain and two biological replicates for the Δ*tanX* strain were performed. For each RNAseq sample a dataset of at least 1.5 Gb (20 million reads) of Illumina-filtered sequence data was obtained. Processing of the RNA seq data and differences in transcript expression between genotypes were analyzed with DESeq2 as described (Niu et al., 2017 and references therein).

### Molecular Techniques and Construction of Gene Deletion Strains

The gene deletion strains used in this study were created using either the split marker method (Arentshorst et al., 2015), or by using the marker-free CRISPR/Cas9 genome editing method (van Leeuwe et al., 2019). For PCR amplifications Phire Hot Start II DNA Polymerase (Thermo Scientific) was used. Amplification and purification of split marker fragments were done as described (Arentshorst et al., 2015) using gene specific primer sets which are listed in Supplementary Table 1. The *tanX* and *tanR* genes were deleted using the split marker method using hygromycin as selection marker. Deletion of *tanX* was confirmed by Southern blot analysis (Supplementary Figure 1) and deletion of *tanR* was verified via diagnostic PCR (Supplementary Figure 2). Deletion of NRRL3_06002 was performed with split marker fragments containing the *Aspergillus oryzae pyrG* marker *(AopyrG)* and putative deletions strain were analyzed via diagnostic PCR (Supplementary Figure 3). Deletion of the six gene clusters that were induced in the Δ*tanX* mutant (Δ*NRRL3_01651-NRRL3_01656*, Δ*NRRL3_02180-NRRL3_02185*, Δ*NRRL3_06147-NRRL3_06150*, Δ*NRRL3_08290-NRRL3_08292*, Δ*NRRL3_09054-NRRL3_09057*, and Δ*NRRL3_10365-NRRL3_10375*) was performed with split marker fragments and putative deletions strain were verified by diagnostic PCR (Supplementary Figures 4–9). Single deletion of NRRL3_08632 and double deletion of NRRL3_08632 and NRRL3_04277 were made by split markers using the *AopyrG* marker and hygromycin marker, respectively (Supplementary Figure 10). Deletion of NRRL3_04659 was performed with split marker fragments and *AopyrG* and proper deletion was verified by diagnostic PCR (Supplementary Figure 11).

To create a strain with all the seventeen putative ring cleavage enzymes deleted (Δ*rce* 17x) from its genome, the marker-free CRISPR/Cas9 genome editing method (van Leeuwe et al., 2019) was used. To increase the chance of successfully creating a double strand break in the gene of interest, two sgRNA targets per gene were designed using the CHOPCHOP web-tool (Labun et al., 2019). For each gene two pFC332-based Cas9-hygR-AMA1 vectors each containing a unique Pro1-sgRNA expression cassette were constructed. The guide RNAs were PCR amplified (Supplementary Table 1) and cloned into pFC332. The sequence of final plasmids was verified by sequencing (Macrogen, The Netherlands).

For each gene deletion, a repair DNA fragment was constructed to allow homologous recombination at the locus of the gene of interest. This repair DNA fragment consists of the 5′ flank and the 3′ flank of the gene, separated by a 23 bp spacer. The flanks of all ring cleavage enzymes were PCR amplified with genomic DNA of N402 as template and primers that are listed in Supplementary Table 1. The overlapping spacer sequence in all PCR products is used in a subsequent fusion PCR to fuse together the 5′ flank and 3′ flank, resulting in the repair DNA fragment. The sizes of the flanks and repair DNA fragments for each ring cleavage enzyme are shown in Supplementary Table 1. Deletion of genes encoding the ring cleavage enzymes was achieved by transforming strain MA234.1 (Δ*ku70*) with plasmids pFC332-gRNA1 and pFC332-gRNA2 (both 2–5 μg DNA) and the repair DNA fragment (1–2 μg DNA). Transformants were purified on MM supplemented with hygromycin, followed by a purification step on MM to allow loss of the hygR-AMA1 pFC332 plasmids. Afterwards the loss of these plasmids was confirmed by growth analyses on MM supplemented with hygromycin and correct integration of the repair DNA was verified by diagnostic PCR. In one transformation two or three genes were deleted simultaneously resulting in the seventeen ring-cleavage enzyme deletion strain MA925.2 (Table 1), confirmed by diagnostic PCR (Supplementary Figure 12).

### Metabolite Analysis

The extracellular metabolite production by parental and mutant *A. niger* strains was performed using a two-step culture; a primary culture generating fungal biomass and a secondary culture to induce protein and metabolite production. For primary culture generation, 100 mL of CM with 2% fructose was inoculated with 2 × 10^6^ spores/mL and incubated for 24 h at 30°C with shaking at 220 rpm. Primary cultures were filtered through with a Buchner funnel lined with Miracloth (Calbiochem). The collected mycelia was rinsed with sterile ddH_2_O and a volume of 5 mL of mycelia was used to start secondary cultures in 250 mL flasks containing 50 mL MM, 1% gallic acid, and 0.1% fructose. Secondary cultures were incubated at 30°C with shaking at 220 rpm. Two mL of culture liquid was harvested at 0, 4, 8, 18, and 24 h. For each time point, the sample was centrifuged at 15 000 × *g* for 15 min. A 100 μL aliquot of supernatant was transferred to a tube with 200 μL cold methanol and left at −20°C overnight. The samples were centrifuged at 15 000 × *g* for 30 min, then a 30 μL aliquot of the supernatant was mixed with 150 μL of 0.1% formic acid. A 10 μL aliquot was analyzed by high resolution liquid chromatography-mass spectrometry using an Agilent 1,260 Infinity II HPLC system (Agilent technologies, Santa Clara, CA, USA) connected in-line to 7 Tesla Thermo-Finnigan LTQ-FT mass spectrometer (Thermo Electron Corporation, San Jose, CA). Chromatography separation of sample components was done using a Synergi Hydro-RP 150 × 2.0 mm, 4 μm column (Phenomenex, Torrence, CA, USA). The solvents used to generate the gradient during reversed-phase separation were 0.1% formic acid in water for Solvent A and 0.1% formic acid in acetonitrile for Solvent B. Solvent flow rate was 300 μL/min and the gradient started at 1% B, increased to 30% B in 6 min, increased to 80% B in 2 min, increased to 90% B in 0.1 min, maintained at 90% for 1 min, decreased to 1% B in 0.1 min and kept at 1% B for 4 min. Column eluate was connected to a Thermo-Finnigan Ion Max electrospray source. Spectra were acquired in negative mode from 60 to 800 m/z at 50,000 resolution at 200 m/z. LC-MS data were processed for ion feature extraction and compound annotation using Compound Discoverer 3.0 (Thermo-Fisher Scientific, Waltham, MA). Compound annotation was done by querying the metabolite databases from ChEBI, ChEMBL, and MoNA using a 5 ppm mass accuracy search window criteria. The relative abundance of selected compounds of interest were compared across samples using extracted chromatogram peak area values calculated by the Xcalibur 2.2. Quan Browser software (Thermo-Fisher Scientific, Waltham, MA).

## Results

### Tannases Are Constitutively Expressed in Putative Repressor Mutant Δ*NRRL3_08276*

**Figure 2:**
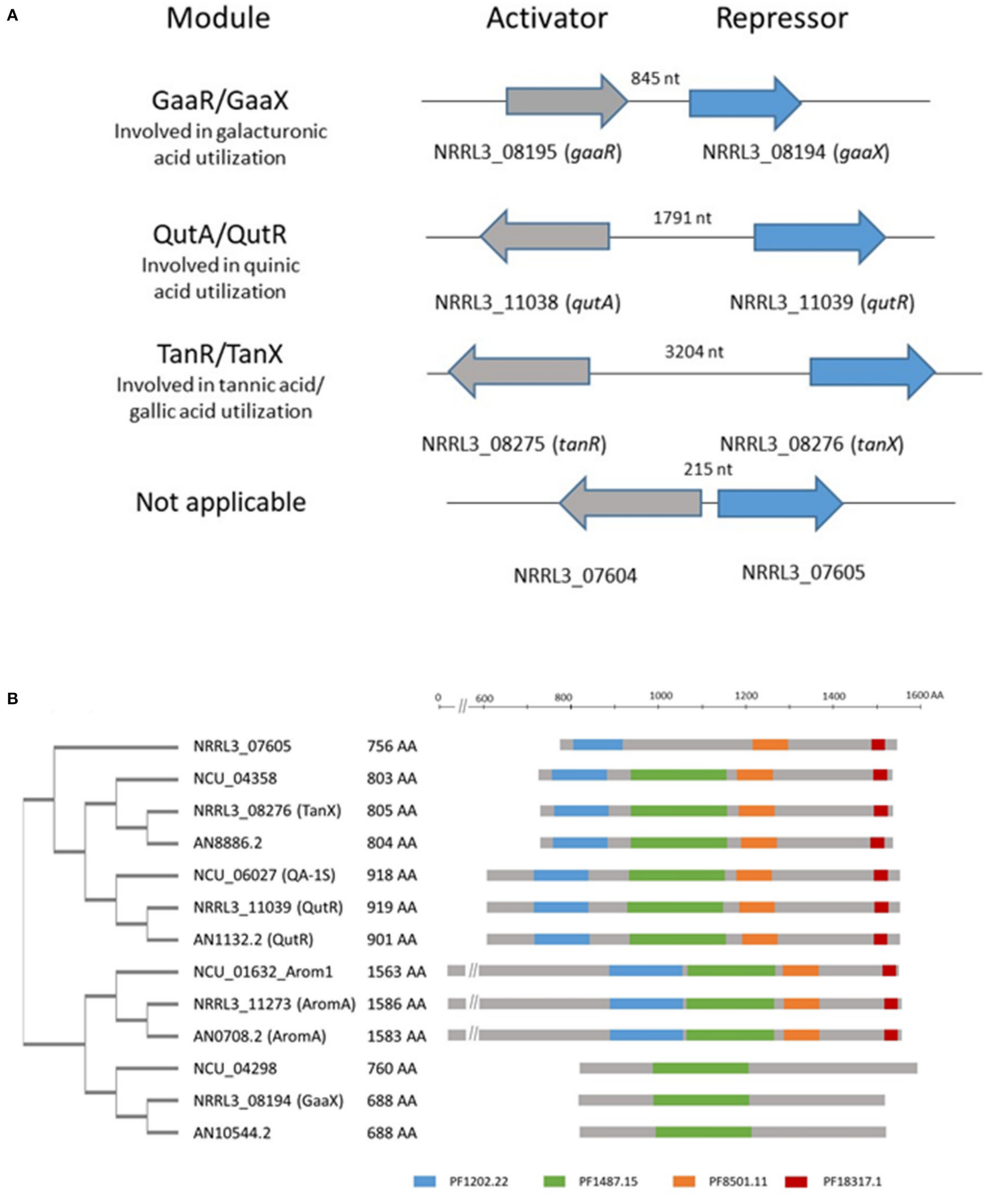
Schematic overview of the genomic organization of activator/repressor modules in the genome of *A. niger* and the presence of PFAM domains in putative repressor proteins. **(A)** Four modules consisting of an activator and a putative repressor protein are present in the genome of *A. niger*. The genes ecoding the activator and repressor proteins are localized adjacent to each other and either expressed in a head to tail orientation (*gaaR/gaaX*) or a potential bidirectional promotor (*qutA/qutR*; *tanR/tanX* and NRRL3_07604/ NRRL3_07605). The length of the bidirectional promoter is indicated. **(B)** Schematic representation of the phylogeny and relevant PFAM domains present in identified putative repressor protein in *A. niger (NRRL3), A. nidulans* (AN), and *N. crassa* (Ncu), compared to AROM. Protein sequences were retrieved using BLASTP with the AromA protein sequence of *A. niger* (NRRL3_11273) as a query against *A. nidulans* (FGSCA4) or *N. crassa* (OR74A). Orthologs of identified repressor in the tree fungal species are indicated by the colored boxes. PFAM searches were performed at https://www.ebi.ac.uk/Tools/pfa/pfamscan/. The unrooted tree was constructed using standard settings in Clustal Omega at https://www.ebi.ac.uk/Tools/msa/clustalo/.

We identified a transcriptional activator-repressor module in *A. niger* which shows similarities in both genomic organization and protein sequence to the activator-repressor modules involved in galacturonic acid or quinic acid utilization (Niu et al., 2017; Figure 2A). The transcriptional activator [NRRL3_08275 (An03g6810)] is a Zn(II)_2_Cys_6_ type transcription factor. The predicted repressor [NRRL3_08276 (An03g06800)] shows homology to repressor proteins that are involved in galacturonic acid ([NRRL3_08194 (GaaX) and quinic acid (NRRL3_11039 (QutR)] utilization and a fourth putative repressor (NRRL3_07605) with unknown function (Niu et al., 2017). The family of four putative repressor proteins in *A. niger* identified previously (Niu et al., 2017) consists of proteins with conserved protein family domains (PFAM domains) that are similarly organized as in the AROM (aromatic biosynthesis) protein. AROM is a conserved and large protein (1,586 amino acids long in *A. niger*) composed of five domains, and the individual domains are required for five enzymatic steps representing the prechorismate shikimate pathway which is required for aromatic amino acid biosynthesis (Duncan et al., 1987). A BLASTP search using AROM1 protein of *A. niger* (NRRL3_11273) as a query identified the four putative repressor proteins in *A. niger*. The homology between AROM and the putative repressor is found in the last three domains of the AROM protein. These last three domains of AROM consist of a shikimate kinase domain (PF01202.22), a 3-dehydroquinate dehydratase domain (PF01487.15) and a shikimate dehydrogenase domain (PF08501.11 and PF18317.1). These domains are also present in the orthologous repressor protein identified in *A. nidulans* and *N. crassa* (Figure 2B). The putative repressor protein (NRRL3_08194) was found in BLASTP searches using AROM as a query, but the PF1202.22, PF8501.11, and PF18317.15 domains in the GaaX paralogs are not recognized in PFAM searches (Figure 2B). In addition, PF01487.15 was not detected in NRRL3_07605.

Loss of function of the galacturonic acid specific repressor protein (GaaX) or the quinic acid specific repressor protein (QutR) results in constitutive activation of expression of genes involved in pectin utilization and galacturonic acid metabolism (Δ*gaaX*) or quinic acid metabolism (Δ*qutR*), respectively (Niu et al., 2017; Ram and Tsang, unpublished data). To identify metabolic pathways which could be controlled by the NRRL3_08275-NRRL3_08276 module, a RNAseq study with deletion mutant of the putative repressor gene [MA586.1 (Δ*NRRL3_08276*)] was performed (see Materials and Methods for details and Supplementary Figure 1 for strain verification). No growth abnormality in terms of radial growth or sporulation was observed for the deletion mutant compared to the parental strain when cultured on minimal medium agar plates with glucose as carbon source (data not shown).

To identify which metabolic pathways are controlled by this regulatory module, RNAseq analysis was carried out. The deletion mutant (Δ*NRRL3_08276*) and its parent (MA234.1), were cultivated in bioreactors and RNA was isolated from exponentially growing cells on fructose medium. Fructose was chosen as a carbon source as it is generally less strong as a carbon catabolite repressing sugar compared to glucose. The specific growth rate (0.217 ± 0.002 g dry weight/kg broth/h) and maximal biomass accumulation (4.289 ± 0.194 g dry weight/kg broth) of the Δ*NRRL3_08276* strain were similar to the growth rate and maximal biomass accumulation (0.214 ± 0.007 g dry weight/kg broth/ h and 4.151 ± 0.134 g dry weight/kg broth) of parental strain MA234.1. The cultures and transcriptomic data of MA234.1 used for comparison have been previously described (Niu et al., 2017). Total RNA was isolated from *A. niger* strain Δ*NRRL3_08276* and MA234.1 when 70% of the maximal biomass was reached. RNAseq analysis was performed to identify genes that were significantly higher expressed in the Δ*NRRL3_08276* mutant than in the parental strain (Supplementary Table 2). A DESeq2 analysis on the transcriptome data was performed to define differentially expressed genes. This analysis indicated that 330 genes displayed significantly higher expression (FC > 2 and FDR < 0.001) (Supplementary Table 2). Among the twenty genes with the largest expression increase in the putative repressor mutant compared to the parental strain (Table 2) are two genes (NRRL3_00063, NRRL3_06367) encoding predicted tannases, suggesting that the NRRL3_08275/NRRL3_08276 module regulates genes involved in tannic acid utilization.

**Table 2:** Top 20 of genes with the highest fold change (FC) in expression between the Δ*tanX* mutant and the parental strain during exponential growth on fructose.

**Transcript**	**Description**	**FC (Dseq^**2**^)**	**FDR**
NRRL3_01655^*a^	O-methyltransferase, COMT-type	5,712.8	0
NRRL3_04659	FAD-binding protein, putative monoxygenase	2,392.2	0
NRRL3_03155	Ser-Thr-rich GPI-anchored protein	452.7	0
NRRL3_01653^*a^	Multicopper oxidase	297.1	3.37E^−150^
NRRL3_08632	Extradiol ring-cleavage dioxygenase	232.7	2.64E^−204^
NRRL3_01656^*a^	Hypothetical protein	189.8	6.69E^−167^
NRRL3_03935	MFS-type transporter	179.7	0
NRRL3_07468	Carboxylesterase	142.5	7.40E^−268^
NRRL3_00063	Tannase/feruloyl esterase family protein	111.3	0
NRRL3_06002	Putative decarboxylase, SnoaL-like domain containing protein	95.8	0
NRRL3_01654^*a^	Prenyltransferase, UbiA family	94.5	3.87E^−77^
NRRL3_06149^*b^	Hypothetical protein	93.1	0
NRRL3_09057^*b^	PAN-1 domain-containing protein	79.8	3.94E^−67^
NRRL3_10195	WW domain-containing protein	65.9	9.40E^−276^
NRRL3_11834	ABC transporter, family 2, CDR-type	65.6	3.69E^−65^
NRRL3_09055^*b^	Hypothetical protein	65.0	1.32E^−58^
NRRL3_09054^*b^	Glucan endo-1,3-alpha-glucosidase	57.7	4.40E^−64^
NRRL3_04136	Guanyl-Specific ribonuclease	51.6	4.17E^−218^
NRRL3_06367	Tannase/feruloyl esterase family protein	47.6	1.59E^−90^
NRRL3_06150^*a^	Isochorismatase family protein	40.7	1.26E^−52^

*FC, Fold change; FDR, False discovery rate.*

*a *Genes belonging to gene cluster comprising NRRL3_01649-NRRL3_01658.*

*b *Genes belonging to gene cluster comprising NRRL3_09054-NRRL3_09057*.

### The Zn(II)_2_Cys_6_ Transcription Factor NRRL3_08275 Is Required for Growth on Tannic Acid and Gallic Acid

**Figure 3:**
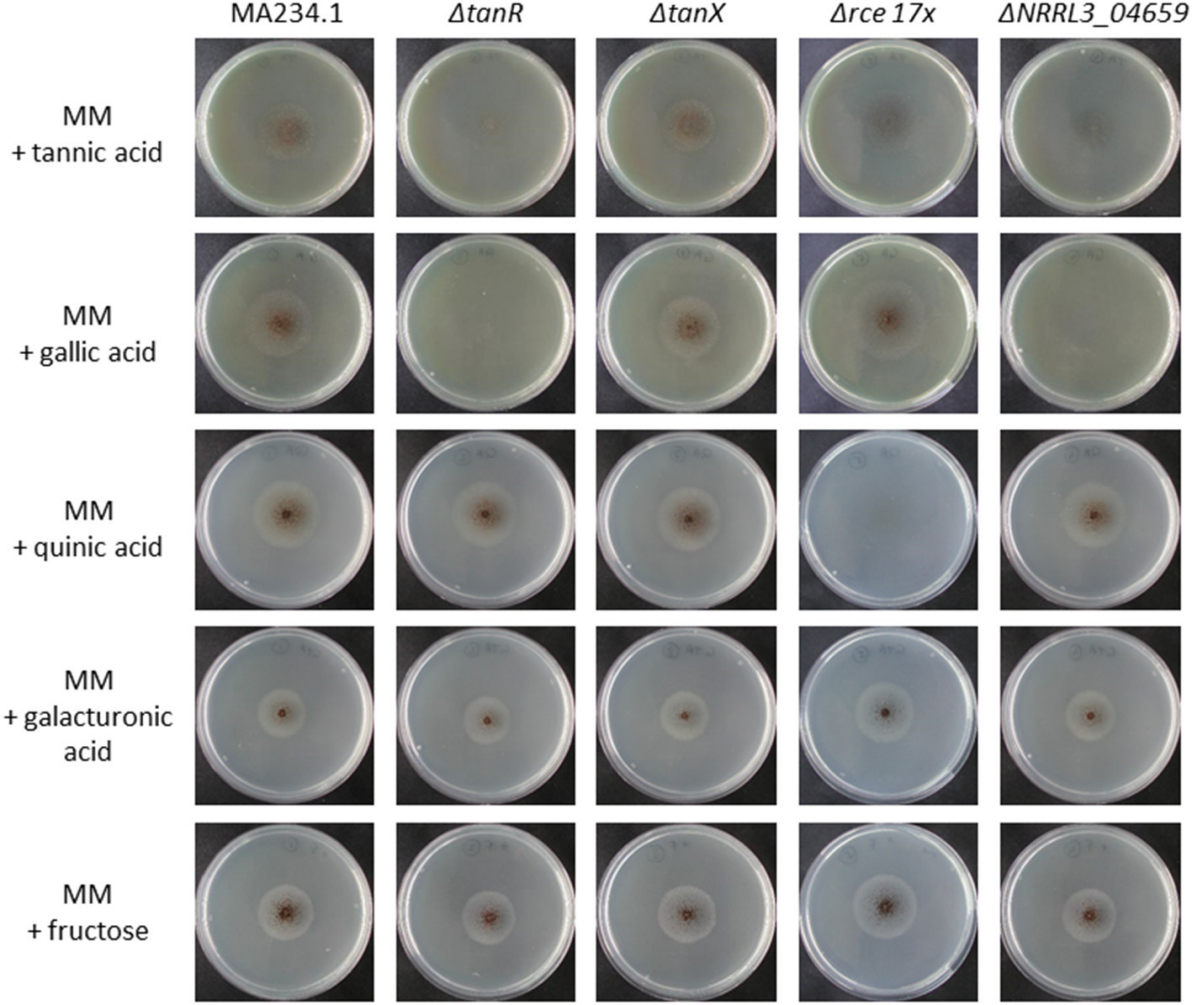
Growth analysis of *A. niger* reference strain (MA234.1), Δ*tanR*, Δ*tanX*, Δ*rce 17x*, and Δ*gacA* (Δ*NRRL3_04659)* on various carbon sources. Spores were point inoculated on minimal medium (MM) supplemented with the indicated carbon source (5 mM). Strains were grown for 5 days at 30°C before pictures were taken.

To test whether the module is indeed related to tannic acid utilization, the Zn(II)_2_Cys_6_ transcriptional activator NRRL3_08275 (An03g6810) was deleted (Supplementary Figure 2). Growth on substrates including tannic acid and its main monomeric constituent gallic acid was analyzed. As shown in Figure 3, growth of Δ*NRRL3_08275* was severely reduced on tannic acid and no growth occurred on gallic acid, confirming that NRRL3_08275 regulates tannic acid degradation. To determine whether the growth defect of Δ*NRRL3_08275* was specific for tannic acid and gallic acid, growth of Δ*NRRL3_08275* on galacturonic acid, quinic acid (Figure 3) and on various aromatics including ferulic acid, p-hydroxybenzoic acid, protochatechuic acid, caffeic acid, vanillic acid, p-coumaric acid, gentisic (2,5-dihydroxybenzoic) acid, salicylic acid, and catechol (Supplementary Figure 13) was tested. Δ*NRRL3_08275* grew on these substrates equally well as the control strain (MA234.1), indicating that the growth defect of Δ*NRRL3_08275* is specifically related to the utilization of tannic acid and its main degradation product gallic acid (Figure 3; Supplementary Figure 13). Growth of the putative repressor knockout (Δ*NRRL3_08276*) was not affected by any of the compounds tested (Figure 3; Supplementary Figure 13). We therefore refer to the Zn(II)_2_Cys_6_ transcriptional activator NRRL3_08275 as TanR and the putative repressor protein NRRL3_08276 as TanX, analogous to the GaaR/GaaX activator-repressor module (Niu et al., 2017). The expression of the *tanR* transcription factor is 4-fold induced in the Δ*tanX* mutant (Supplementary Table 2). This upregulation of the *tanR* transcription factor in the absence of the putative repressor suggests that *tanR* itself is also controlled by the *tanR/tanX* module and *tanR* can upregulate itself.

### A Putative Gallic Acid Decarboxylase That Is Induced in the Δ*tanX* Strain Is Not Required for Growth on Gallic Acid

Having established that the TanR/TanX module is likely to control degradation of tannic acid by inducing the expression of predicted tannases and the utilization of gallic acid, we further examined the gene expression pattern of the *tanX* mutant compared to its parental strain for genes possibly involved in catabolism of gallic acid (Table 2). The first candidate gene was identified based on homology to Agdc1p, a gallic acid decarboxylase involved in the utilization of gallic acid in the *yeast B. adeninivorans*. BLASTp searches using Agdc1p identified a possible ortholog, NRRL3_06002, which showed the highest similarity (51% identity and 69% similarity) to Agdc1p. The expression of NRRL3_06002 is strongly induced in the Δ*tanX* mutant compared to the parental strain (FC = 95.9; FDR < 0.001) (Table 2; Supplementary Table 2). A deletion mutant of NRRL3_06002 was constructed, verified via diagnostic PCR (Supplementary Figure 3), and its growth on tannic acid and gallic acid analyzed. The Δ*NRRL3_06002* mutant grew normally on tannic acid and gallic acid, indicating that this gene is not required for growth on gallic acid. This result therefore indicates that gallic acid catabolism in *A. niger* is different from that of *B. adeninivorans*, possibly via redundant or alternative pathways.

### Gene Clusters That Are Upregulated in the Δ*tanX* Strain Are Dispensable for Growth on Gallic Acid

Six gene clusters (NRRL3_01651-NRRL3_01656; NRRL3_02180-NRRL3_02185; NRRL3_06147-NRRL3_06150; NRRL3_08290-NRRL3_08292; NRRL3_09054-NRRL3_09057; NRRL3_10365-NRRL3_10375) were identified because the genes in these clusters were upregulated in the Δ*tanX* mutant compared to the parental strain (Supplementary Table 3). Since in some cases genes encoding enzymes involved in catabolic pathways are clustered (e.g., quinic acid or galacturonic acid), we examined the possibility that genes in these clusters were involved in tannic acid or gallic utilization by deleting these gene clusters. Deletion mutants [Δ*NRRL3_01651-NRRL3_01656* (MA829.1); Δ*NRRL3_02180-NRRL3_02185* (MA852.1); Δ*NRRL3_06147-NRRL3_06150* (TS10.1); Δ*NRRL3_08290-NRRL3_08292* (MA857.2); Δ*NRRL3_09054-NRRL3_09057* (MA963.4); Δ*NRRL3_10365-NRRL3_10375* (MA860.2)] were verified by diagnostic PCR (Supplementary Figures 4–9) and tested for growth on gallic acid and tannic acid. All deletion mutants grew normally on gallic acid and tannic acid indicating that these gene clusters are not exclusively required for tannic acid degradation and gallic acid utilization (data not shown). Since the gene clusters were deleted individually, it cannot be formally excluded that these gene clusters have redundant functions in gallic acid catabolism.

### Intra- or Extradiol Ring Cleavage Enzymes in the *A. niger* Genome Are Not Required for Growth on Gallic Acid

**Figure 4:**
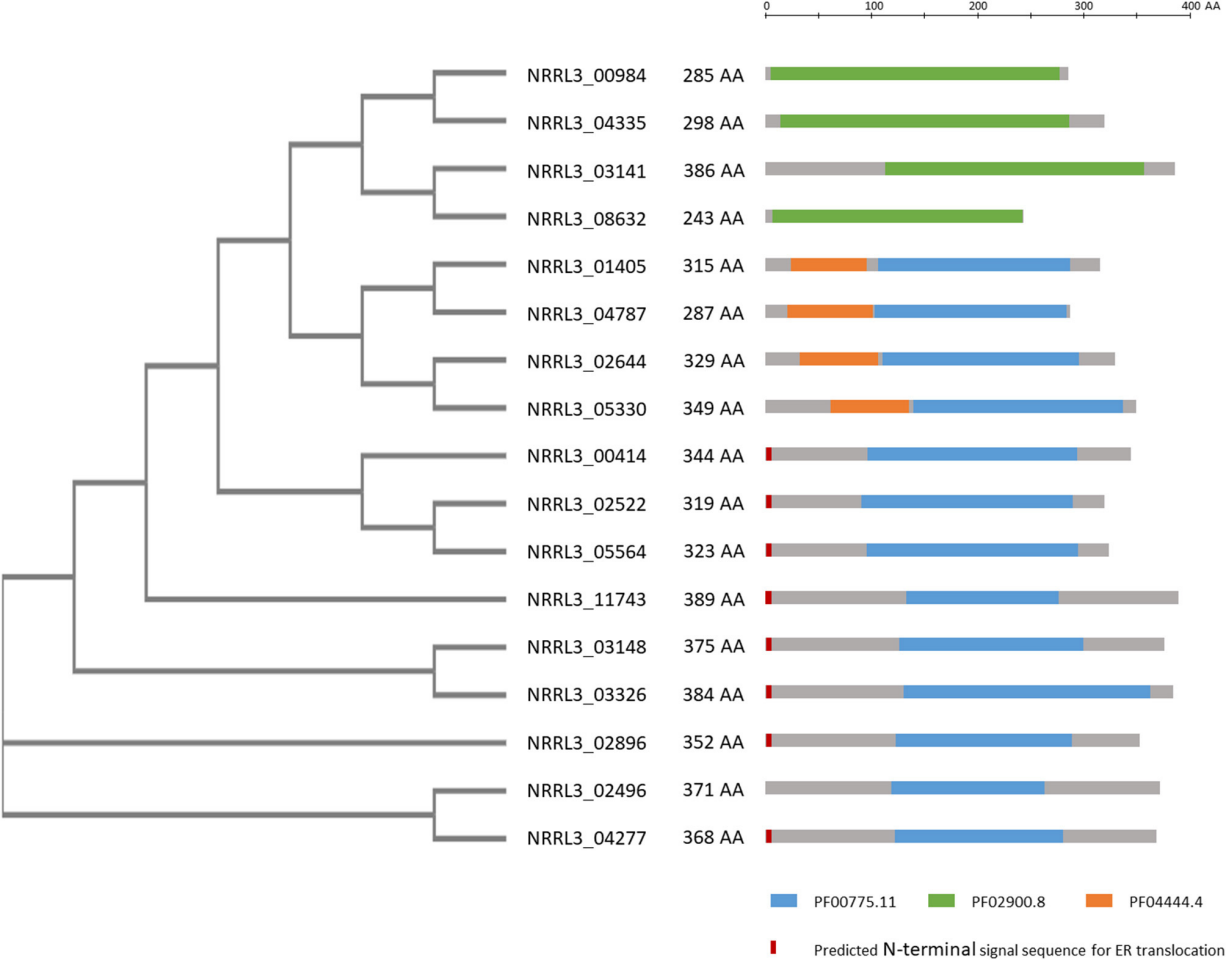
Overview of phylogeny and PFAM domains of 17 ring cleavage enzymes in the genome of *A. niger*. Phylogenetic relationship of the proteins, their length in amino acids are given. Gray bars indicate the enzymes with the colored bars indicating PFAM domains present on the enzyme. PFAM domains include: Blue: PF00775.11: dioxygenase, Green: PF02900.8: catalytic LigB subunit of aromatic ring-opening dioxygenase, Orange: PF04444.4: catechol dioxygenase N-terminus. In red/orange the presence of a predicted signal sequence is given. PFAM predictions were taken from https://mycocosm.jgi.doe.gov/pages/search-for-genes.jsf?organism=Aspni_NRRL3_1. Signal sequence predictions were carried out using SignalP-5.0 at http://www.cbs.dtu.dk/services/SignalP/. The unrooted tree was constructed using standard settings in Clustal Omega at https://www.ebi.ac.uk/Tools/msa/clustalo/.

In a further attempt to identify genes encoding proteins involved in gallic acid utilization, the role of putative ring cleavage enzymes, identified to be relevant for growth on gallic acid in bacteria, was addressed. Two putative ring cleavage enzymes encoded by NRRL3_08632 and NRRL3_04277 were 232 and 38-fold induced in the Δ*tanX* mutant (Table 2; Supplementary Table 4). Single and double deletion mutants were constructed, verified (Supplementary Figure 10), and tested for growth on gallic acid and tannic acid. The growth on gallic acid of either single mutants or the double mutant was not affected (data not shown) indicating that the ring cleavage of gallic acid does not exclusively depend on these two ring cleavage enzymes, leaving the possibility that other ring cleavage enzymes might take over their function. To address this possibility, we searched the *A. niger* genome and identified seventeen genes encoding potential ring cleavage enzymes based on BlastP results in combination with PFAM domain analysis. These seventeen proteins were selected because of the presence of PF00775.11, PF02900.8, and/or PF04444.4 domains which are related to a dioxygenase_C domain, the catalytic LigB subunit of aromatic ringopening dioxygenase domain and/or the Dioxygenase_N domain, respectively. The domain structure in combination with the phylogenetic relationship of these proteins are shown in Figure 4. The expression of these seventeen genes was analyzed in the Δ*tanX*/parental expression data set and is shown in Supplementary Table 4. To address the possibility that functionally redundant ring cleavage enzymes occur within this group of seventeen enzymes, we constructed an *A. niger* strain in which all seventeen genes encoding ring cleavage enzymes were deleted (Supplementary Figure 12). Growth of the 17-fold ring cleavage enzyme mutant on gallic acid or tannic acid was not impaired compared to the parental strain, indicating that none of the ring cleavage enzymes is required for gallic acid utilization (Figure 3). Growth of the 17-fold deletion strain on quinic acid is abolished (Figure 3) which is due to deletion of NRRL3_01405 (Sgro, Arentshorst, Ram and Tsang, manuscript in preparation).

### A FAD Domain-Containing Monooxygenase Is Required for Growth on Gallic Acid

In the transcriptome data the second most induced gene (Table 2) is NRRL3_04659. This gene encodes a FAD domain-containing monooxgenase with sequence similarity to salicylate hydroxylase (NahG) of *P. putida*. As ring cleavage has also been reported to be mediated by monooxygenases and because of its strong induction in the Δ*tanX* mutant, a knockout strain for this gene was made and verified by diagnostic PCR (Supplementary Figure 11). Growth assays on various aromatic compounds revealed that the Δ*NRRL3_04659* mutant does not grow on gallic acid and shows strongly reduced growth on tannic acid (Figure 3). Growth on galacturonic acid, quinic acid, and aromatic compounds such as ferulic acid, p-hydroxybenzoic acid, protocatechuic acid, caffeic acid, vanillic acid, p-coumaric acid, gentisic acid, salicylic acid, and catechol was not affected by deletion of NRRL3_04659 (Figure 3; Supplementary Figure 13), implying that this monooxygenase gene, which we called *gacA* for gallic acid catabolism, is required and specific for the initial ring cleavage of gallic acid.

Growing the Δ*gacA* on agar plates containing 2.5 mM fructose as a carbon source and comparing the growth on agar plates containing 2.5 mM fructose and 25 mM gallic acid revealed that the addition of gallic acid reduced growth of the Δ*gacA* mutant (as well as the Δ*tanR* mutant) compared to the parental strain (Supplementary Figure 14). This preliminary result indicates that the ability to metabolize gallic acid also contributes to the detoxification of gallic acid.

**Figure 5:**
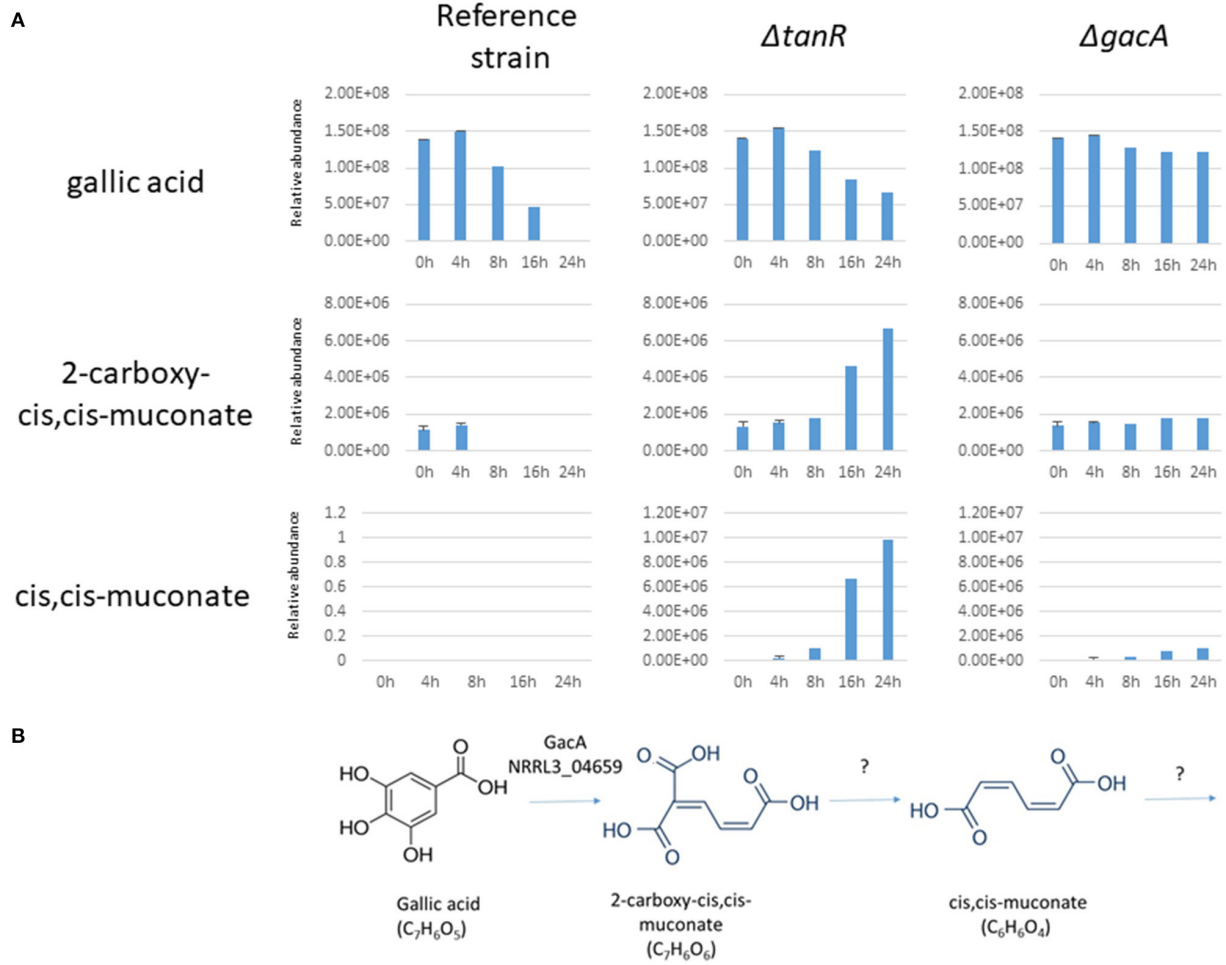
**(A)** Extracellular metabolites analysis of *A. niger* strains grown after transfer to 1% gallic acid/0.1% fructose. *A. niger* strains MA234.1 (reference strain), the Δ*tanR* mutant, and the Δ*gacA (NRRL3_04659)* mutant were pregrown in CM supplemented with fructose for 24 h, mycelium was washed and transferred MM supplemented with 1% gallic acid/0.1% fructose for the indicated times. Most abundant extracellular metabolites (gallic acid, 2-carboxy-cis,cis-muconate and cis,cis-muconate) were detected and their relative abundance over time course (0, 4, 8, 16, and 24 h after transfer) were analyzed as described in Materials and Methods. **(B)** Proposed first steps in catabolism of gallic acid in *A. niger* based on the accumulation of intermediates in the Δ*tanR* mutant.

Further indications that GacA is required for ring cleavage of gallic acid was obtained by extracellular metabolite analysis. The parental strain (MA234.1) and the Δ*gacA* strains (TS6.4) were grown on fructose/gallic acid medium for 18 and 24 h. The Δ*tanR* strain was also included in the same experiment. Extracellular metabolites present in the medium after 18 and 24 h were detected by LC-MS-MS as described in the Materials and Methods. The analysis showed that levels of gallic acid in the medium of the Δ*gacA* remain the same during the time course (Figure 5A). In contrast, gallic acid levels in the parental strain (MA234.1) dropped over time, and after 24 h of growth hardly any gallic acid remained. Interestingly, in the Δ*tanR* mutant, gallic acid levels dropped over time and other metabolites which could be identified as muconate and 2-carboxy-*cis,cis*-muconate were accumulating in the medium. Possibly 2-carboxy-*cis,cis*-muconate and muconate are intermediates in the catabolism of gallic acid and the expression of the gene encoding the enzyme acting on 2-carboxy-*cis*_*cis*-muconate is no longer induced in the Δ*tan*R mutant (Figure 5B). This could result in accumulation of 2-carboxy-*cis*-*cis*-muconate if *gacA* was still expressed to sufficient levels to convert gallic acid into 2-carboxy-*cis*-*cis*-muconate.

## Discussion

In the genome of *A. niger* over 440 different Zn(II)_2_Cys_6_ transcription factors can be found (Ram, unpublished). Most of the Zn(II)_2_Cys_6_ transcription factors are expected to be transcriptional activators and are responsible for the controlled expression of their target genes in response to the right inducer. The functional characterization of this large family of Zn(II)_2_Cys_6_ transcription factors is still very limited and even less is known about the detailed activation mechanisms. Among the large set of Zn(II)_2_Cys_6_ transcription factors in *A. niger*, we previously noticed the presence of four Zn(II)_2_Cys_6_ transcription factors, each of their encoding gene is co-localized in the genome with a gene encoding a putative repressor protein (Niu et al., 2017). One of the Zn(II)_2_Cys_6_ transcription factors that is clustered with a repressor is the transcriptional activator involved in quinic acid utilization (QutA). This system has been studied in detail both in *A. nidulans* and in *N. crassa* (Giles et al., 1991). The repressor protein (QutR) shows significant sequence similarity toward the C-terminal half of the AROM protein (Figure 1B). AROM is a large multidomain protein and individual domains are involved in five enzymatic steps in the prechorismate pathway needed for the biosynthesis of aromatic amino acids (Duncan et al., 1987). The working hypothesis is that the repressor is a catalytically inactive protein that can still recognize or bind to the inducer. Apart from the activator-repressor module that regulates quinic acid utilization, a second module (GaaR/GaaX) has been functionally characterized; this module is involved in the regulation of genes involved in (poly)-galacturonic acid (pectin) degradation and utilization (Niu et al., 2017). In the current study, we show that a third activator and putative repressor module (TanR and TanX) is required for tannic acid degradation and gallic acid utilization. The function of the fourth activator-repressor module (NRRL3_07604 and NRRL3_07605) is currently unknown. All four putative repressor proteins show low sequence similarity toward each other and to the AROM1 protein. The mechanism by which the putative repressor and activator interact is unknown, but it has been suggested that an inducer molecule binds to the putative repressor protein which results in dissociation of the repressor from its conjugate transcriptional activator. The release of the repressor is suggested to result in an active transcription factor (Giles et al., 1991; Niu et al., 2017). The notion that all repressors show sequence similarity with AROM indicates that these putative repressors share a common evolutionary origin, and how these conserved modules have evolved is an intriguing question for further research.

The transcriptomic analysis of the putative repressor knockout mutant (Δ*tanX*) revealed a number of genes under the control of the TanR/TanX module. By analogy with the studies we carried out with the repressor mutant related to galacturonic acid utilization (the Δ*gaaX* mutant), we expected target genes of the module to be expressed in higher levels in the repressor knockout mutant compared to the parental strain. In this study, our interest was drawn to the genes involved in the utilization of gallic acid which is not well-characterized in fungi. One of the most highly induced genes, NRRL3_06002, encodes a putative decarboxylase with high sequence similarity to a gallic acid decarboxylase from the yeast *B. adeninivorans* (Agdc1). In *B. adeninivorans*, gallic acid is degraded to pyrogallol before further degradation via 2-hydroxymuconic acid and oxalocrotonate (Meier et al., 2017). *A. niger* seems to have a different, or at least an alternative, catabolic pathway since the deletion of NRRL3_06002 did not result in a gallic acid non-utilizing phenotype. The possibility of redundancy of a gallic acid decarboxylase was examined and three homologs of NRRL3_06002 (NRRL3_06896, NRRL3_07601 and NRRL3_07467) were identified, but none of them showed a >2 fold higher induction in the Δ*tanX* mutant compared to the parental strain. The expression values of the three homologs were relatively low in either the reference strain or the Δ*tanX* mutant (Supplementary Table 2; Table 2), making it unlikely that one of these genes is responsible for taking over the function of NRRL3_06002. In agreement with that, the metabolite analysis in the parental strain, the Δ*tanR* mutant, and the Δ*gacA* (Δ*NRRL3_04659*) mutant did not reveal the presence of pyrogallol, indicating that the degradation pathway of gallic acid does not involve direct decarboxylation from gallic acid to pyrogallol as an intermediate as in the case of *B. adeninivorans* (Meier et al., 2017), *A. oryzae* (Guo et al., 2014) and some bacterial species (Jiménez et al., 2013; Sonia et al., 2017).

Analysis of the top 20 genes that were fold-change wise the highest expressed in the Δ*tanX* strain compared to the parental strain identified several genes that were clustered to each other (Table 2). Therefore, the entire transcriptome data was analyzed for gene clusters that were significantly upregulated in the Δ*tanX* mutant compared to the parental strain. This resulted in the identification of six clusters consisting of three genes or more that were induced in the Δ*tanX* mutant (Supplementary Table 3). These gene clusters were individually deleted from the genome, but the deletion mutants were still able to grow as well as the parental strains on gallic acid indicating that none of the genes in these clusters is exclusively required for gallic acid utilization. Two gene clusters (cluster NRRL3_10365-NRRL3_10375 and cluster NRRL3_02180-NRRL3_02185) contain a polyketide synthase encoding gene and a non-ribosomal peptide encoding gene (NRRL3_10375 and NRRL3_02185, respectively), which suggests that these clusters could be related to secondary metabolite production.

The molecular mechanism on how the putative TanX repressor protein controls the activity of the TanR transcription factor is not known. By analogy with the modules regulating quinic acid utilization in *A. nidulans* (QutA/QutR) and *N. crassa* (QA-1F/QA-1S) and galacturonic acid utilization in *A. niger* (GaaR/GaaX) a highly speculative model can be envisioned. In this model, we expect the transcriptional repressor and the transcriptional activator to be bound to each other under non-inducing conditions, leading to an inactive transcription factor complex. Upon the presence of an inducer, we expect binding of the inducer to the putative repressor protein as discussed previously (Niu et al., 2017), leading to the dissociation of the putative repressor from the transcription factor/repressor complex. Dissociation of the putative repressor from the transcription factor is expected to result in an active transcription factor. For quinic acid utilization, quinic acid or a metabolic derivative from quinic acid (Huiet and Giles, 1986) and in the case of galacturonic acid utilization 2-keto-3-deoxygalactonate (Alazi et al., 2017) are indicated to be this inducer, while for the gallic acid utilization pathway this inducer is currently unknown. In addition to regulation of the activity of TanR at the protein level, the TanR transcription factor is also transcriptionally regulated as we observed a 4-fold induction of *tanR* expression in the Δ*tanX* mutant, indicating a positive feedback loop in that TanR can also induce its own expression. It should be noted that the current model of interactions between the inducer, putative repressor and transcriptional activator are highly speculative and needs to be tested in future studies.

Another intriguing result of our study is that in *A. niger* potential ring cleavage enzymes (both intra- and extradiol acting) are not involved in gallic acid utilization. Even though two potential ring cleavage enzymes (NRRL3_04277 and NRRL3_08632) were highly induced in the Δ*tanX* strain, their simultaneous deletion did not result in a growth defect on gallic acid. To rule out the possibility that other ring cleavage enzymes would take over each other's function in gallic acid utilization, all seventeen genes annotated to encode potential ring cleavage enzymes were deleted using marker-free CRISPR-CAS9 genome editing. The 17-fold mutant was still able to grow on gallic acid, indicating an alternative way of ring-opening. The proposed function of two intradiol-acting dioxygenases based on their biochemical characterization (Semana and Powlowski, 2019) was confirmed in the deletion strains. NRRL3_01405 *(prcA)* was predicted to encode a protocatechuate-3,4-dioxygenase and deletion of NRRL3_01405 resulted in the inability to grow on protocatechuic acid (Lubbers et al., 2019c; Sgro et al., unpublished), explaining the inability of the 17-fold deletion mutant to grow on quinic acid (Figure 3). Likewise, NRRL3_04787 was predicted to encode a catechol-1,2-dioxygenase and deletion of NRRL3_04787 resulted in the inability to grow on catechol (Lubbers et al., 2021; Arentshorst and Ram, unpublished data). These results also show that the catabolism of gallic acid does not involve a pathway that includes protocatechuic acid or catechol as an intermediate.

The identification of the transcription factors controlling tannase expression also offers the possibility to optimize and increase the production of tannases. Tannases are biotechnologically interesting enzymes that are applied both in the clarification of wines, fruit juices and coffee-flavored drinks as well as in the production of gallic acid (Dhiman et al., 2018, Beniwal et al., 2013). As shown in this study, constitutive expression of certain tannases can be achieved by deleting the gene encoding the putative repressor protein *tanX*. Based on analogy with the GaaR/GaaX system, one can predict that overexpression of the gene encoding the TanR transcription factor will result in inducer-independent production of tannases, just as overexpression of *gaaR* led to inducer-independent expression of pectinases (Alazi et al., 2018).

## Data Availability Statement

The datasets presented in this study can be found in online repositories. RNA seq data regarding the parental strains (MA234.1) were previously deposited in the Short Read Archive under accession number SRP078415 (Niu et al., 2017). The RNA seq data regarding the Δ*tanX* mutant have been deposited in the Short Read Archive under accession number PRJNA713317. Metabolomics data have been deposited to the EMBL-EBI MetaboLights database (DOI: 10.1093/nar/gkz1019, PMID:31691833) with the identifier MTBLS2667.

## Author Contributions

MA: conceptualization, investigation, validation, data analysis, methodology, and visualization. MF: investigation, data analysis, methodology, investigation, and data analysis. M-CM: investigation and data analysis. IR: RNA sequencing and data analysis. TS and JD: investigation and data analysis. ED: conceptualization, data analysis, and manuscript draft writing. JP: conceptualization and data analysis. PP: conceptualization and manuscript draft writing. AT: conceptualization, data analysis, and funding. AR: conceptualization, data analysis, original draft writing, funding acquisition, and supervision. All authors contribute to the writing and agree to be accountable for the content of the work.

## Conflict of Interest

The authors declare that the research was conducted in the absence of any commercial or financial relationships that could be construed as a potential conflict of interest.

## Supplementary Material

Supplementary Figure 1 Southern blot analysis to verify *tanX::hygR* deletion in MA234.1 **(A)** Schematic representation of the *tanX* locus and the *tanX::hygR* locus in the deletion strain. DNA fragments expected to hybridize with the probe after digestion of genomic DNA with *Eco*RI are indicated. **(B)** Genomic DNA of putative Δ*tanX::hygR* transformants and the recipient strain MA234.1 was analyzed. MA586.1 was selected for further analysis.

Supplementary Figure 2 Diagnostic PCR to verify *tanR::hygR* deletion in MA234.1 **(A)** Schematic representation of the *tanX::hygR* locus in the deletion strain. Diagnostic PCR is performed using primer set 08275_P5f and hygP5r (5′ PCR) and primer set hygP2f and 08275_P6r (3′ PCR). The locations where the primers anneal are indicated. **(B)** PCR reactions were performed with genomic DNA of putative *tanR::hygR* transformants as template and PCR products were analyzed using gel electrophoresis. MA514.1 was selected for further analysis.

Supplementary Figure 3 Diagnostic PCR to verify *NRRL3_06002::AopyrG* deletion in MA169.4 **(A)** Schematic representation of the *NRRL3_06002* locus in the wild type (wt) strain and the *NRRL3_06002::AopyrG* locus in the deletion strain. Diagnostic PCR is performed using primer set 06002_P9f and 06002_P10r (wt 5′ PCR), primer set 06002_P7f and 06002_P8r (wt 3′ PCR), primer set 06002_P9f and AopyrGP16r (Δ 5′ PCR) and primer set AopyrGP17f and 06002_P8r (Δ 3′ PCR). The location where the primers anneal is indicated. **(B)** PCR reactions were performed with genomic DNA of a putative *NRRL3_06002::AopyrG* transformant and genomic DNA of wt strain N402 as template and PCR products were analyzed using gel electrophoresis. MA831.1 was used for further analysis.

Supplementary Figure 4 Diagnostic PCR to verify *NRRL3_01651-56::AopyrG* deletion in MA169.4. **(A)** Schematic representation of the *NRRL3_01651-56* locus in the wild type (wt) strain and the *NRRL3_01651-56::AopyrG* locus in the deletion strain. Diagnostic PCR is performed using primer set 01651_P5f and 01651_P6r (wt 5′ PCR), primer set 01656_P7f and 01656_P8r (wt 3′ PCR), primer set 01651_P5f and AopyrGP16r (Δ 5′ PCR) and primer set AopyrGP17f and 01656_P8r (Δ 3′ PCR). The location where the primers anneal is indicated. **(B)** PCR reactions were performed with genomic DNA of twelve putative *NRRL3_01651-56::AopyrG* transformants and genomic DNA of wt strain N402 as template and PCR products were analyzed using gel electrophoresis. MA829.1 was used for further analysis.

Supplementary Figure 5 Diagnostic PCR to verify *NRRL3_02180-85::AopyrG* deletion in MA169.4. **(A)** Schematic representation of the *NRRL3_02180-85* locus in the wild type (wt) strain and the *NRRL3_02180-85::AopyrG* locus in the deletion strain. Diagnostic PCR is performed using primer set 02185_P5f and 02185_P6r (wt 5′ PCR), primer set 02180_P7f and 02180_P8r (wt 3′ PCR), primer set 02185_P5f and AopyrGP16r (Δ 5′ PCR) and primer set AopyrGP17f and 02180_P8r (Δ 3′ PCR). The location where the primers anneal is indicated. **(B)** PCR reactions were performed with genomic DNA of three putative *NRRL3_02180-85::AopyrG* transformants and genomic DNA of wt strain N402 as template and PCR products were analyzed using gel electrophoresis. MA852.1 was used for further analysis.

Supplementary Figure 6 Diagnostic PCR to verify *NRRL3_06147-50::AopyrG* deletion in MA169.4. **(A)** Schematic representation of the *NRRL3_06147-50* locus in the wild type (wt) strain and the *NRRL3_06147-50::AopyrG* locus in the deletion strain. Diagnostic PCR is performed using primer set 06147_P5f and 06147_P6r (wt 5′ PCR), primer set 06150_P7f and 06150_P8r (wt 3′ PCR), primer set 06147_P5f and AopyrGP16r (Δ 5′ PCR) and primer set AopyrGP17f and 06150_P8r (Δ 3′ PCR). The location where the primers anneal is indicated. **(B)** PCR reactions were performed with genomic DNA of four putative *NRRL3_06147-50::AopyrG* transformants and genomic DNA of wt strain N402 as template and PCR products were analyzed using gel electrophoresis. TS10.1 was used for further analysis.

Supplementary Figure 7 Diagnostic PCR to verify *NRRL3_08290-92::AopyrG* deletion in MA169.4. **(A)** Schematic representation of the *NRRL3_08290-92* locus in the wild type (wt) strain and the *NRRL3_08290-92::AopyrG* locus in the deletion strain. Diagnostic PCR is performed using primer set 08290_P5f and 08290_P6r (wt 5′ PCR), primer set 08292_P7f and 08292_P8r (wt 3′ PCR), primer set 08290_P5f and AopyrGP16r (Δ 5′ PCR) and primer set AopyrGP17f and 08292_P8r (Δ 3′ PCR). The location where the primers anneal is indicated. **(B)** PCR reactions were performed with genomic DNA of two putative *NRRL3_08290-92::AopyrG* transformants and genomic DNA of wt strain N402 as template and PCR products were analyzed using gel electrophoresis. MA857.2 was used for further analysis.

Supplementary Figure 8 Diagnostic PCR to verify *NRRL3_09054-57::AopyrG* deletion in MA169.4. **(A)** Schematic representation of the *NRRL3_09054-57* locus in the wild type (wt) strain and the *NRRL3_09054-57::AopyrG* locus in the deletion strain. Diagnostic PCR is performed using primer set 09054_P3f and 09054_P4r (wt 5′ PCR), primer set 09057_P3f and 09057_P4r (wt 3′ PCR), primer set 09054_P3f and AopyrGP16r (Δ 5′ PCR) and primer set AopyrGP17f and 09057_P4r (Δ 3′ PCR). The location where the primers anneal is indicated. **(B)** PCR reactions were performed with genomic DNA of four putative *NRRL3_09054-57::AopyrG* transformants and genomic DNA of wt strain N402 as template and PCR products were analyzed using gel electrophoresis. MA963.4 was used for further analysis.

Supplementary Figure 9 Diagnostic PCR to verify *NRRL3_10365-75::AopyrG* deletion in MA169.4. **(A)** Schematic representation of the *NRRL3_10365-75* locus in the wild type (wt) strain and the *NRRL3_10365-75::AopyrG* locus in the deletion strain. Diagnostic PCR is performed using primer set 10365_P5f and 10365_P6r (wt 5′ PCR), primer set 10375_P9f and 10375_P10r (wt 3′ PCR), primer set 10365_P5f and AopyrGP16r (Δ 5′ PCR) and primer set AopyrGP17f and 10375_P10r (Δ 3′ PCR). The location where the primers anneal is indicated. **(B)** PCR reactions were performed with genomic DNA of a putative *NRRL3_10365-75::AopyrG* transformant and genomic DNA of wt strain N402 as template and PCR products were analyzed using gel electrophoresis. MA860.2 was used for further analysis.

Supplementary Figure 10 Diagnostic PCR to verify *NRRL3_08632::AopyrG and NRRL3_04277::hygR* deletion mutants. **(A)** Schematic representation of the *NRRL3_08632* locus in the wild type (wt) strain and the *NRRL3_08632::AopyrG* locus in the deletion strain. Diagnostic PCR is performed using primer set 08632_P5f and 08632_P6r (wt 5′ PCR), primer set 08632_P7f and 08632_P8r (wt 3′ PCR), primer set 08632_P5f and AopyrGP16r (Δ 5′ PCR) and primer set AopyrGP17f and 08632_P8r (Δ 3′ PCR). The location where the primers anneal is indicated. **(B)** PCR reactions were performed with genomic DNA of a putative *NRRL3_08632::AopyrG* transformant and genomic DNA of wt strain N402 as template and PCR products were analyzed using gel electrophoresis. TS2.1 was used for further analysis. **(C)** Schematic representation of the *NRRL3_04277* locus in the wild type (wt) strain and the *NRRL3_04277::hygR* locus in the deletion strain. Diagnostic PCR is performed using primer set 04277_P5f and 04277_P6r (wt 5′ PCR), primer set 04277_P7f and 04277_P8r (wt 3′ PCR), primer set 04277_P5f and hygP5r (Δ 5′ PCR) and primer set hygP2f and 04277_P8r (Δ 3′ PCR). The location where the primers anneal is indicated. **(D)** PCR reactions were performed with genomic DNA of a putative *NRRL3_04277::hygR* transformant and genomic DNA of wt strain N402 as template and PCR products were analyzed using gel electrophoresis. MA835.1 was used for further analysis.

Supplementary Figure 11 Diagnostic PCR to verify *NRRL3_04659::AopyrG* deletion in MA169.4. **(A)** Schematic representation of the *NRRL3_04659* locus in the wild type (wt) strain and the *NRRL3_04659::AopyrG* locus in the deletion strain. Diagnostic PCR is performed using primer set 04659_P20f and 04659_P21r (wt 5′ PCR), primer set 04659_P22f and 04659_P23r (wt 3′ PCR), primer set 04659_P20f and AopyrGP16r (Δ 5′ PCR) and primer set AopyrGP17f and 04659_P23r (Δ 3′ PCR). The location where the primers anneal is indicated. **(B)** PCR reactions were performed with genomic DNA of a putative *NRRL3_04659::AopyrG* transformant and genomic DNA of wt strain N402 as template and PCR products were analyzed using gel electrophoresis. TS6.4 was used for further analysis.

Supplementary Figure 12 Diagnostic PCR to verify deletion of 17 putative ring cleavage enzymes in MA234.1. **(A)** Schematic representation of a ring cleavage enzyme (RCE) locus in the wild type (wt) strain and the RCE locus in the deletion strain. The location where the primers anneal is indicated. **(B)** For each RCE deletion, a diagnostic PCR is performed using the primer sets shown in the table. Predicted lengths of PCR products for both the wt locus and the deletion locus are also shown. **(C)** PCR reactions were performed with genomic DNA of two putative transformants with a deletion of the 17 RCEs and genomic DNA of wt strain N402 as template and PCR products were analyzed using gel electrophoresis. MA925.2 was used for further analysis.

Supplementary Figure 13 Growth analysis of *A. niger* reference strain (MA234.1), Δ*tanR*, Δ*tanX*, Δ*rce 17x*, and Δ*NRRL3_04659* on various carbon sources. Spores were point inoculated on minimal medium (MM) supplemented with the indicated carbon source (5 mM). Strains were grown for 5 days at 30°C before pictures were taken.

Supplementary Figure 14 Growth analysis of *A. niger* reference strain (MA234.1), Δ*tanR*, and Δ*gacA strains* on 2.5 mM fructose (left), 2.5 mM fructose and 25 mM gallic acid (middle), or 25 mM gallic acid (right). Spores were point inoculated on minimal medium (MM) supplemented with the indicated carbon source. Strains were grown for 3 days at 30°C before pictures were taken.

Supplementary Table 1 Overview of the primers used in this study.

Supplementary Table 2 TPM expression values.

Supplementary Table 3 Gene clusters higher expressed in Δ*tanX*.

Supplementary Table 4 Expression values of genes encoding ring cleavages enzymes.
