# Supplementary material for: Identification of a Conserved Transcriptional Activator-Repressor Module Controlling the Expression of Genes Involved in Tannic Acid Degradation and Gallic Acid Utilization in Aspergillus niger
Source: Front Fungal Biol. 2021 May 25;2:681631. doi: 10.3389/ffunb.2021.681631 (PMC10512348; doi:10.3389/ffunb.2021.681631)
Supplement: Supplementary Figure 1 — Southern blot analysis to verify tanX::hygR deletion in MA234.1 (A) Schematic representation of the tanX locus and the tanX::hygR locus in the deletion strain. DNA fragments expected to hybridize with the probe after digestion of genomic DNA with EcoRI are indicated. (B) Genomic DNA of putative ΔtanX::hygR transformants and the recipient strain MA234.1 was analyzed. MA586.1 was selected for further analysis. [file Data_Sheet_1.DOCX]

**Supplemental Figure 1**


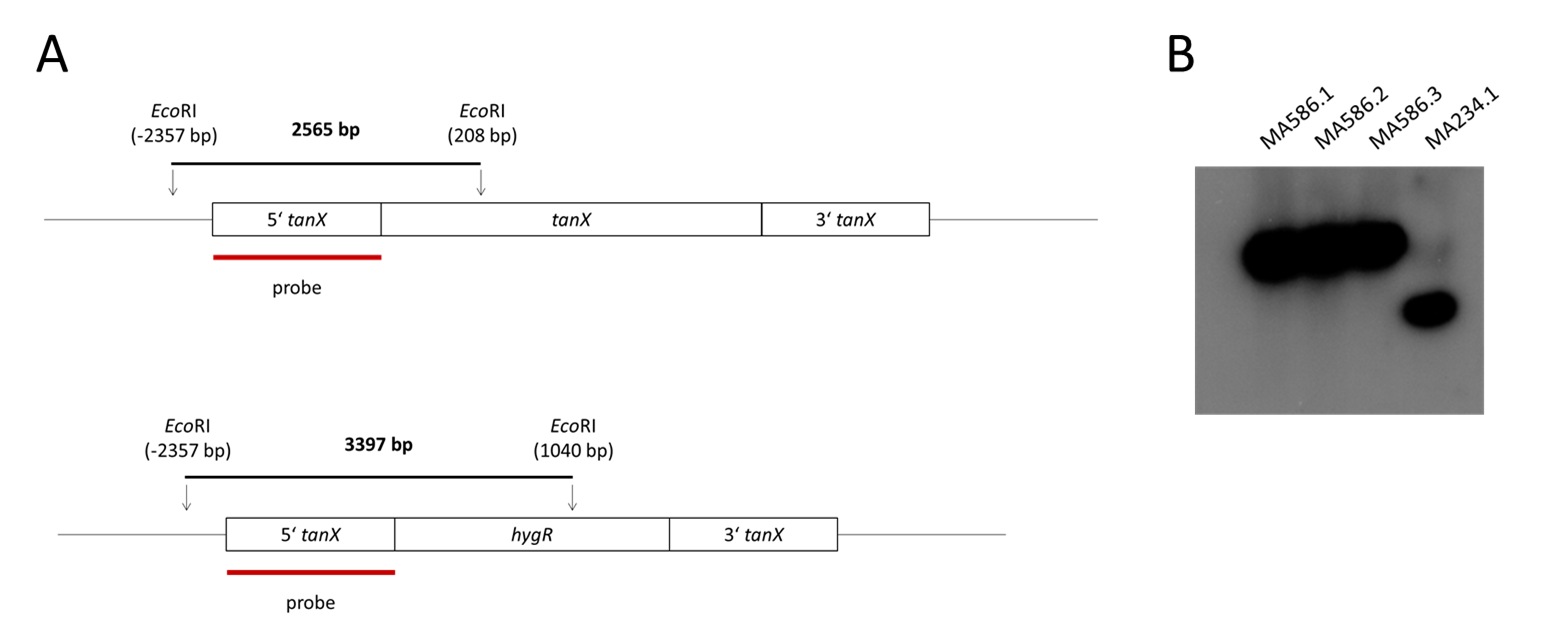


Supplemental Figure 1. Southern blot analysis to verify *tanX::hygR* deletion in MA234.1 A) Schematic representation of the *tanX* locus and the *tanX::hygR* locus in the deletion strain. DNA fragments expected to hybridize with the probe after digestion of genomic DNA with *Eco*RI are indicated. B) Genomic DNA of putative *ΔtanX::hygR* transformants and the recipient strain MA234.1 was analyzed. MA586.1 was selected for further analysis.
