# Supplementary material for: Identification of a Conserved Transcriptional Activator-Repressor Module Controlling the Expression of Genes Involved in Tannic Acid Degradation and Gallic Acid Utilization in Aspergillus niger
Source: Front Fungal Biol. 2021 May 25;2:681631. doi: 10.3389/ffunb.2021.681631 (PMC10512348; doi:10.3389/ffunb.2021.681631)
Supplement: Supplementary Figure 2 — Diagnostic PCR to verify tanR::hygR deletion in MA234.1 (A) Schematic representation of the tanX::hygR locus in the deletion strain. Diagnostic PCR is performed using primer set 08275_P5f and hygP5r (5′ PCR) and primer set hygP2f and 08275_P6r (3′ PCR). The locations where the primers anneal are indicated. (B) PCR reactions were performed with genomic DNA of putative tanR::hygR transformants as template and PCR products were analyzed using gel electrophoresis. MA514.1 was selected for further analysis. [file Data_Sheet_2.DOCX]

**Supplemental Figure 2**


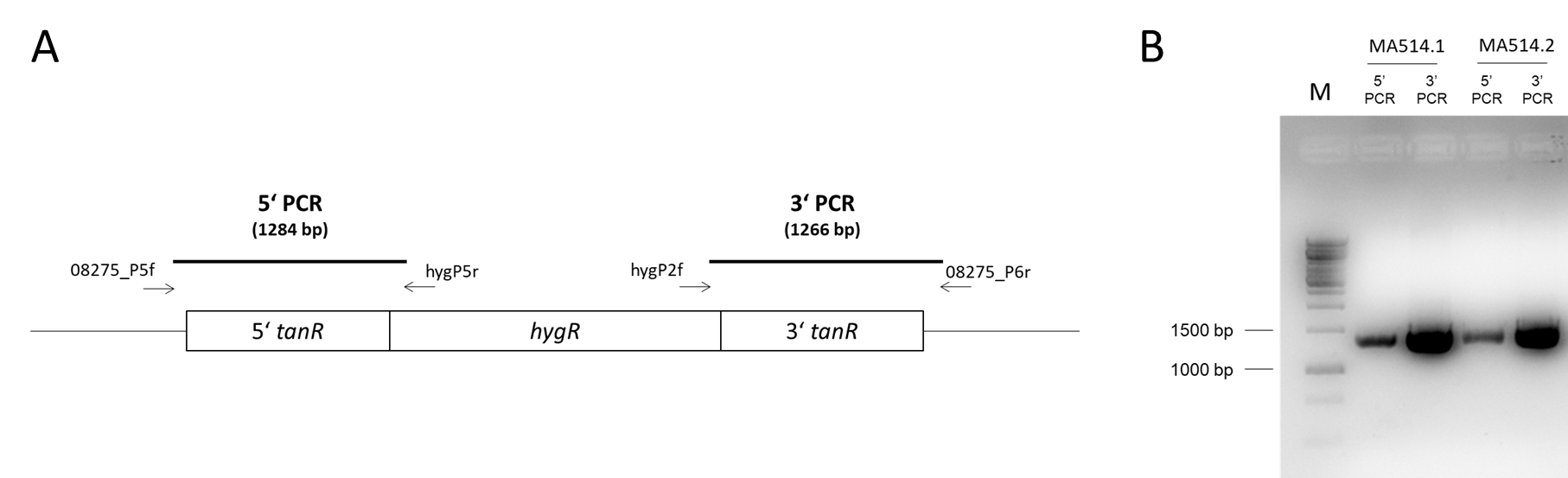


Supplemental Figure 2. Diagnostic PCR to verify *tanR::hygR* deletion in MA234.1 A) Schematic representation of the *tanX::hygR* locus in the deletion strain. Diagnostic PCR is performed using primer set 08275_P5f and hygP5r (5’ PCR) and primer set hygP2f and 08275_P6r (3’ PCR). The locations where the primers anneal are indicated. B) PCR reactions were performed with genomic DNA of putative *tanR::hygR* transformants as template and PCR products were analyzed using gel electrophoresis. MA514.1 was selected for further analysis.
