# Supplementary material for: Identification of a Conserved Transcriptional Activator-Repressor Module Controlling the Expression of Genes Involved in Tannic Acid Degradation and Gallic Acid Utilization in Aspergillus niger
Source: Front Fungal Biol. 2021 May 25;2:681631. doi: 10.3389/ffunb.2021.681631 (PMC10512348; doi:10.3389/ffunb.2021.681631)
Supplement: Supplementary Figure 8 — Diagnostic PCR to verify NRRL3_09054-57::AopyrG deletion in MA169.4. (A) Schematic representation of the NRRL3_09054-57 locus in the wild type (wt) strain and the NRRL3_09054-57::AopyrG locus in the deletion strain. Diagnostic PCR is performed using primer set 09054_P3f and 09054_P4r (wt 5′ PCR), primer set 09057_P3f and 09057_P4r (wt 3′ PCR), primer set 09054_P3f and AopyrGP16r (Δ 5′ PCR) and primer set AopyrGP17f and 09057_P4r (Δ 3′ PCR). The location where the primers anneal is indicated. (B) PCR reactions were performed with genomic DNA of four putative NRRL3_09054-57::AopyrG transformants and genomic DNA of wt strain N402 as template and PCR products were analyzed using gel electrophoresis. MA963.4 was used for further analysis. [file Data_Sheet_8.DOCX]

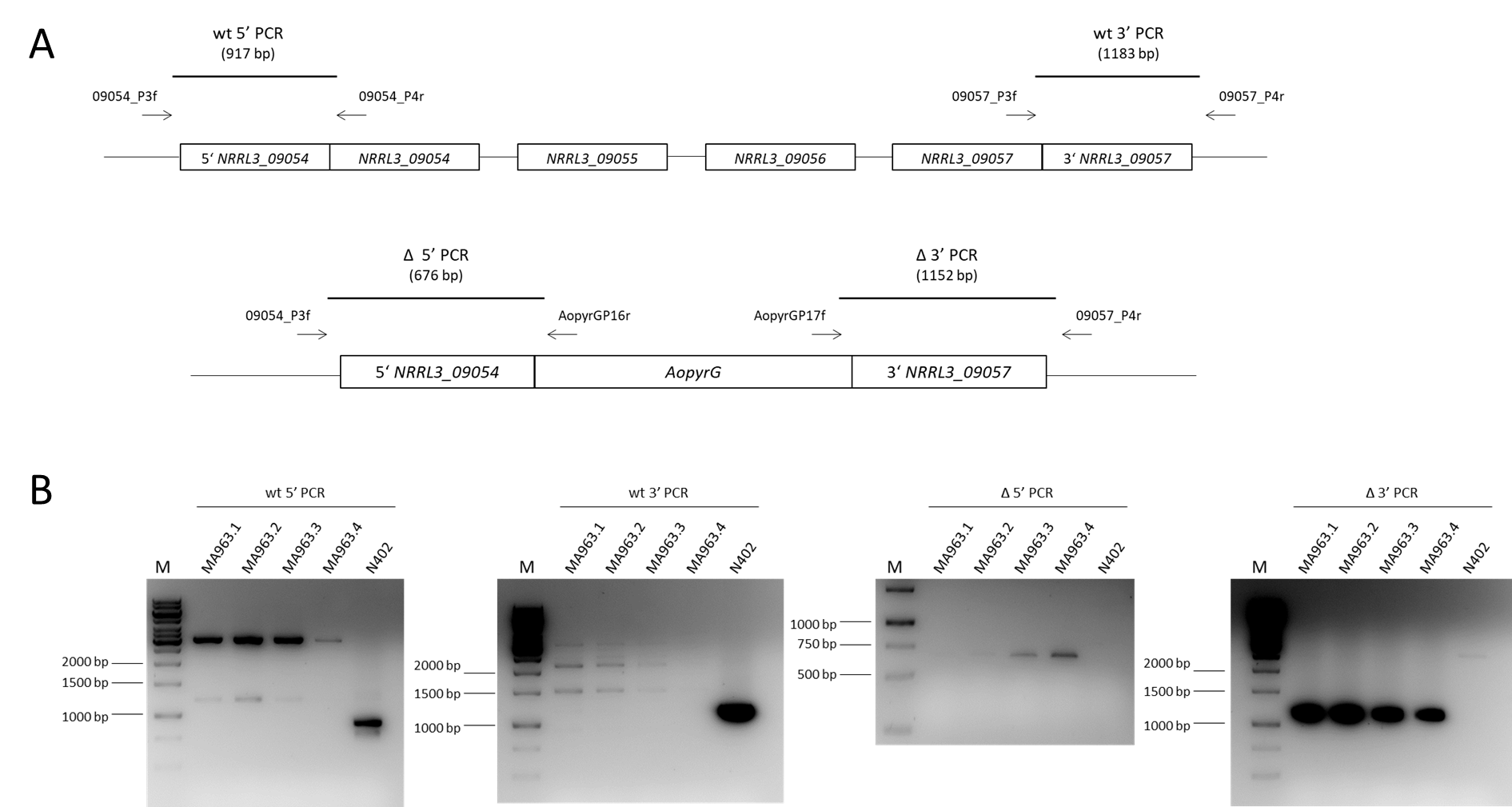


Supplemental Figure 8. Diagnostic PCR to verify *NRRL3_09054-57::AopyrG* deletion in MA169.4

A) Schematic representation of the *NRRL3_09054-57* locus in the wild type (wt) strain and the *NRRL3_09054-57::AopyrG* locus in the deletion strain. Diagnostic PCR is performed using primer set 09054_P3f and 09054_P4r (wt 5’ PCR), primer set 09057_P3f and 09057_P4r (wt 3’ PCR), primer set 09054_P3f and AopyrGP16r (Δ 5’ PCR) and primer set AopyrGP17f and 09057_P4r (Δ 3’ PCR). The location where the primers anneal is indicated. B) PCR reactions were performed with genomic DNA of four putative *NRRL3_09054-57::AopyrG* transformants and genomic DNA of wt strain N402 as template and PCR products were analyzed using gel electrophoresis. MA963.4 was used for further analysis.
