# Supplementary material for: Identification of a Conserved Transcriptional Activator-Repressor Module Controlling the Expression of Genes Involved in Tannic Acid Degradation and Gallic Acid Utilization in Aspergillus niger
Source: Front Fungal Biol. 2021 May 25;2:681631. doi: 10.3389/ffunb.2021.681631 (PMC10512348; doi:10.3389/ffunb.2021.681631)
Supplement: Supplementary Figure 9 — Diagnostic PCR to verify NRRL3_10365-75::AopyrG deletion in MA169.4. (A) Schematic representation of the NRRL3_10365-75 locus in the wild type (wt) strain and the NRRL3_10365-75::AopyrG locus in the deletion strain. Diagnostic PCR is performed using primer set 10365_P5f and 10365_P6r (wt 5′ PCR), primer set 10375_P9f and 10375_P10r (wt 3′ PCR), primer set 10365_P5f and AopyrGP16r (Δ 5′ PCR) and primer set AopyrGP17f and 10375_P10r (Δ 3′ PCR). The location where the primers anneal is indicated. (B) PCR reactions were performed with genomic DNA of a putative NRRL3_10365-75::AopyrG transformant and genomic DNA of wt strain N402 as template and PCR products were analyzed using gel electrophoresis. MA860.2 was used for further analysis. [file Data_Sheet_9.DOCX]

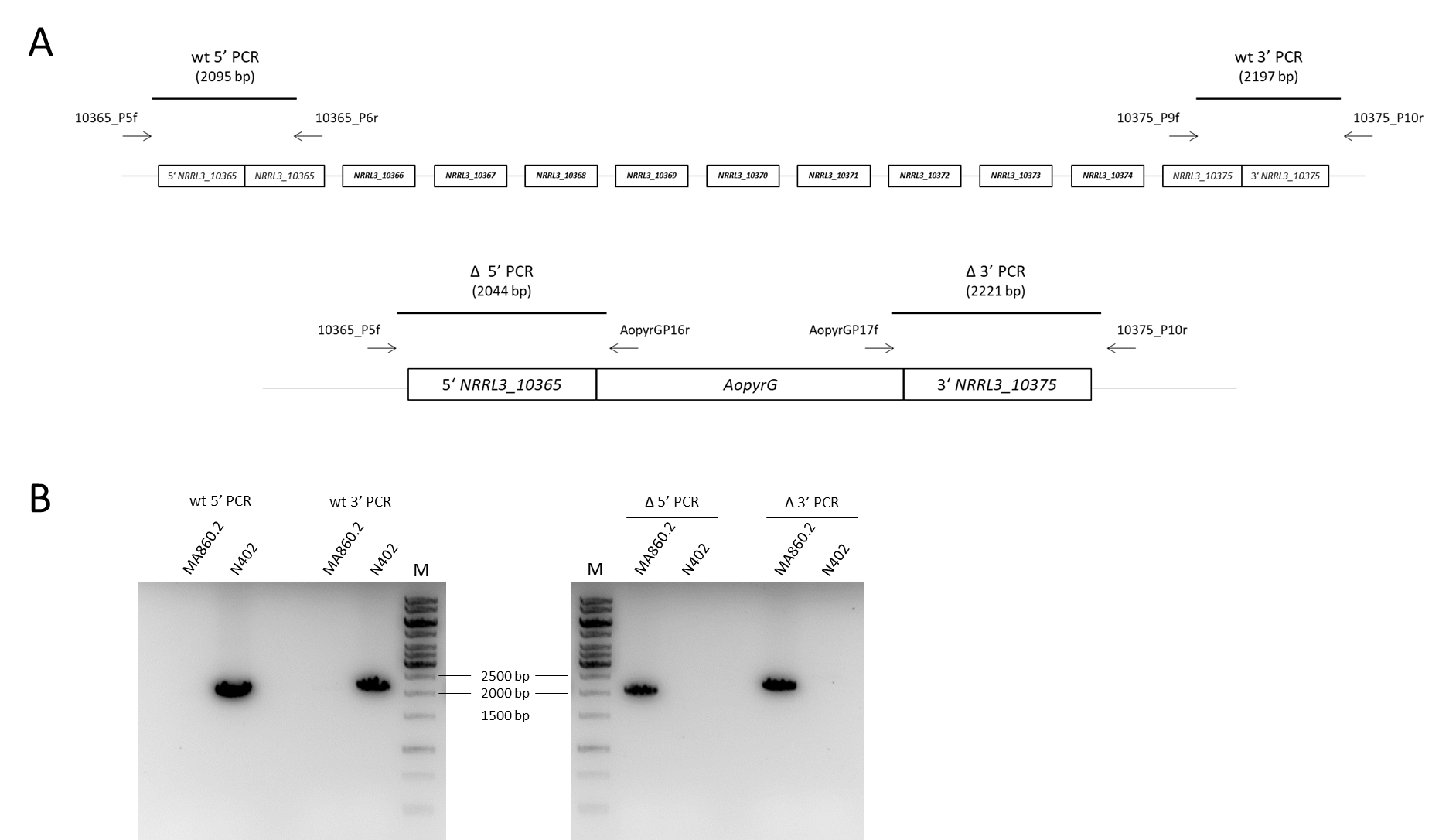


Supplemental Figure 9. Diagnostic PCR to verify *NRRL3_10365-75::AopyrG* deletion in MA169.4. A) Schematic representation of the *NRRL3_10365-75* locus in the wild type (wt) strain and the *NRRL3_10365-75::AopyrG* locus in the deletion strain. Diagnostic PCR is performed using primer set 10365_P5f and 10365_P6r (wt 5’ PCR), primer set 10375_P9f and 10375_P10r (wt 3’ PCR), primer set 10365_P5f and AopyrGP16r (Δ 5’ PCR) and primer set AopyrGP17f and 10375_P10r (Δ 3’ PCR). The location where the primers anneal is indicated. B) PCR reactions were performed with genomic DNA of a putative *NRRL3_10365-75::AopyrG* transformant and genomic DNA of wt strain N402 as template and PCR products were analyzed using gel electrophoresis. MA860.2 was used for further analysis.
