# Supplementary material for: Identification of a Conserved Transcriptional Activator-Repressor Module Controlling the Expression of Genes Involved in Tannic Acid Degradation and Gallic Acid Utilization in Aspergillus niger
Source: Front Fungal Biol. 2021 May 25;2:681631. doi: 10.3389/ffunb.2021.681631 (PMC10512348; doi:10.3389/ffunb.2021.681631)
Supplement: Supplementary Figure 10 — Diagnostic PCR to verify NRRL3_08632::AopyrG and NRRL3_04277::hygR deletion mutants. (A) Schematic representation of the NRRL3_08632 locus in the wild type (wt) strain and the NRRL3_08632::AopyrG locus in the deletion strain. Diagnostic PCR is performed using primer set 08632_P5f and 08632_P6r (wt 5′ PCR), primer set 08632_P7f and 08632_P8r (wt 3′ PCR), primer set 08632_P5f and AopyrGP16r (Δ 5′ PCR) and primer set AopyrGP17f and 08632_P8r (Δ 3′ PCR). The location where the primers anneal is indicated. (B) PCR reactions were performed with genomic DNA of a putative NRRL3_08632::AopyrG transformant and genomic DNA of wt strain N402 as template and PCR products were analyzed using gel electrophoresis. TS2.1 was used for further analysis. (C) Schematic representation of the NRRL3_04277 locus in the wild type (wt) strain and the NRRL3_04277::hygR locus in the deletion strain. Diagnostic PCR is performed using primer set 04277_P5f and 04277_P6r (wt 5′ PCR), primer set 04277_P7f and 04277_P8r (wt 3′ PCR), primer set 04277_P5f and hygP5r (Δ 5′ PCR) and primer set hygP2f and 04277_P8r (Δ 3′ PCR). The location where the primers anneal is indicated. (D) PCR reactions were performed with genomic DNA of a putative NRRL3_04277::hygR transformant and genomic DNA of wt strain N402 as template and PCR products were analyzed using gel electrophoresis. MA835.1 was used for further analysis. [file Data_Sheet_10.DOCX]

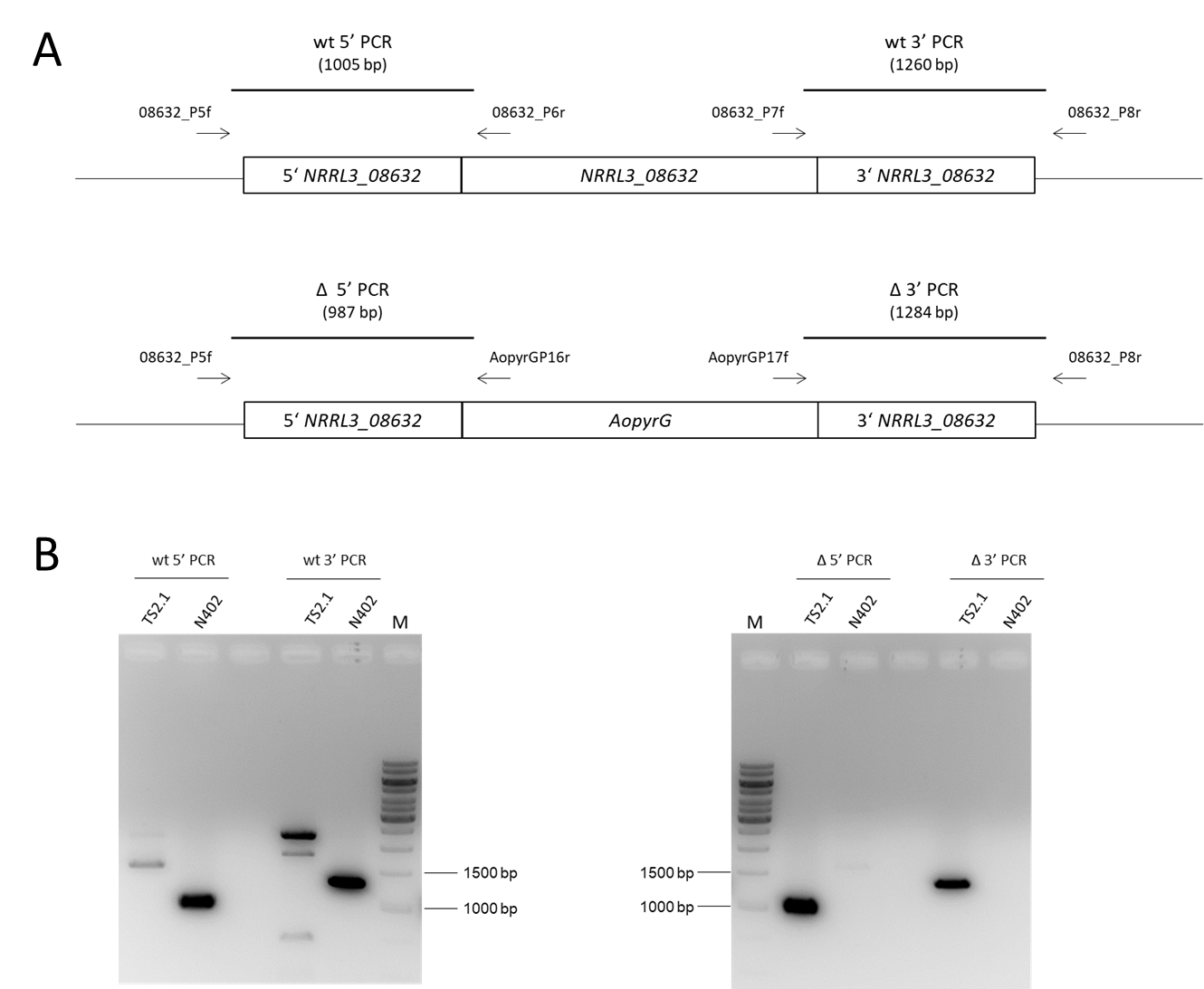


**
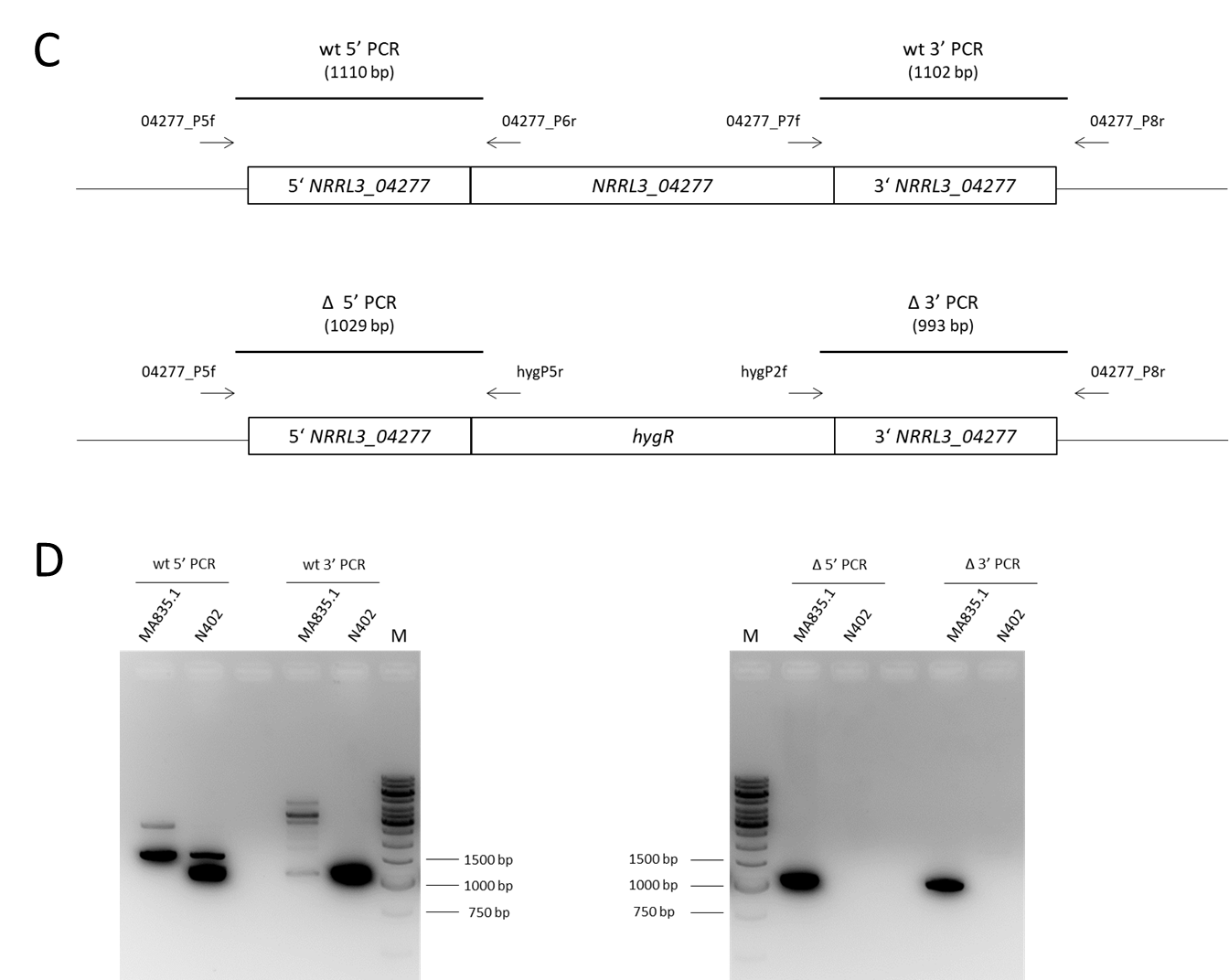
**

Supplemental Figure 10. Diagnostic PCR to verify *NRRL3_08632::AopyrG and NRRL3_04277::hygR* deletion mutants*.* A) Schematic representation of the *NRRL3_08632* locus in the wild type (wt) strain and the *NRRL3_08632::AopyrG* locus in the deletion strain. Diagnostic PCR is performed using primer set 08632_P5f and 08632_P6r (wt 5’ PCR), primer set 08632_P7f and 08632_P8r (wt 3’ PCR), primer set 08632_P5f and AopyrGP16r (Δ 5’ PCR) and primer set AopyrGP17f and 08632_P8r (Δ 3’ PCR). The location where the primers anneal is indicated. B) PCR reactions were performed with genomic DNA of a putative *NRRL3_08632::AopyrG* transformant and genomic DNA of wt strain N402 as template and PCR products were analyzed using gel electrophoresis. TS2.1 was used for further analysis. C) Schematic representation of the *NRRL3_04277* locus in the wild type (wt) strain and the *NRRL3_04277::hygR* locus in the deletion strain. Diagnostic PCR is performed using primer set 04277_P5f and 04277_P6r (wt 5’ PCR), primer set 04277_P7f and 04277_P8r (wt 3’ PCR), primer set 04277_P5f and hygP5r (Δ 5’ PCR) and primer set hygP2f and 04277_P8r (Δ 3’ PCR). The location where the primers anneal is indicated. D) PCR reactions were performed with genomic DNA of a putative *NRRL3_04277::hygR* transformant and genomic DNA of wt strain N402 as template and PCR products were analyzed using gel electrophoresis. MA835.1 was used for further analysis.
