# Supplementary material for: Identification of a Conserved Transcriptional Activator-Repressor Module Controlling the Expression of Genes Involved in Tannic Acid Degradation and Gallic Acid Utilization in Aspergillus niger
Source: Front Fungal Biol. 2021 May 25;2:681631. doi: 10.3389/ffunb.2021.681631 (PMC10512348; doi:10.3389/ffunb.2021.681631)
Supplement: Supplementary Figure 12 — Diagnostic PCR to verify deletion of 17 putative ring cleavage enzymes in MA234.1. (A) Schematic representation of a ring cleavage enzyme (RCE) locus in the wild type (wt) strain and the RCE locus in the deletion strain. The location where the primers anneal is indicated. (B) For each RCE deletion, a diagnostic PCR is performed using the primer sets shown in the table. Predicted lengths of PCR products for both the wt locus and the deletion locus are also shown. (C) PCR reactions were performed with genomic DNA of two putative transformants with a deletion of the 17 RCEs and genomic DNA of wt strain N402 as template and PCR products were analyzed using gel electrophoresis. MA925.2 was used for further analysis. [file Data_Sheet_12.DOCX]

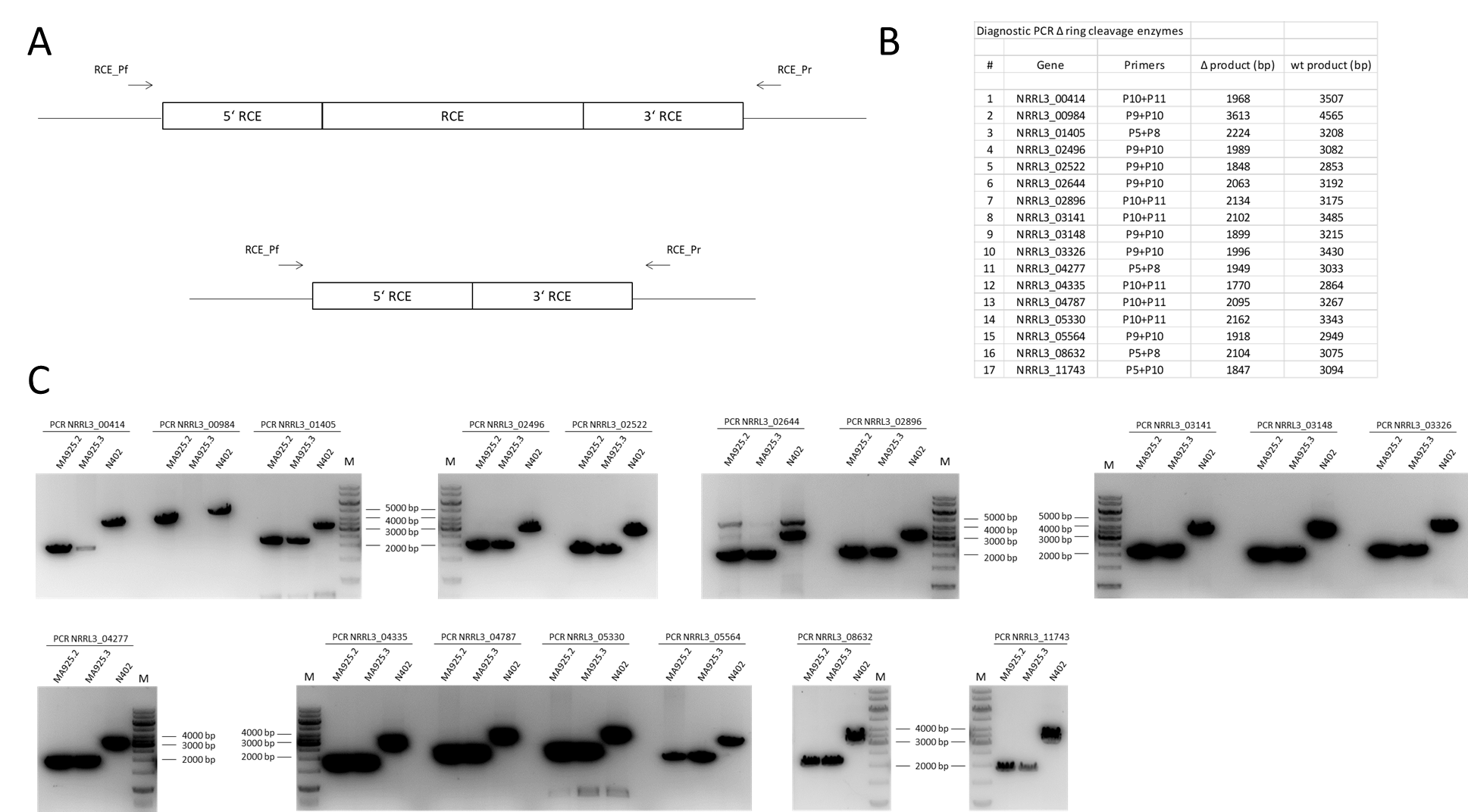


Supplemental Figure 12. Diagnostic PCR to verify deletion of 17 putative ring cleavage enzymes in MA234.1. A) Schematic representation of a ring cleavage enzyme (RCE) locus in the wild type (wt) strain and the RCE locus in the deletion strain. The location where the primers anneal is indicated. B) For each RCE deletion, a diagnostic PCR is performed using the primer sets shown in the table. Predicted lengths of PCR products for both the wt locus and the deletion locus are also shown. C) PCR reactions were performed with genomic DNA of two putative transformants with a deletion of the 17 RCEs and genomic DNA of wt strain N402 as template and PCR products were analyzed using gel electrophoresis. MA925.2 was used for further analysis.
