# Supplementary material for: Identification of a Conserved Transcriptional Activator-Repressor Module Controlling the Expression of Genes Involved in Tannic Acid Degradation and Gallic Acid Utilization in Aspergillus niger
Source: Front Fungal Biol. 2021 May 25;2:681631. doi: 10.3389/ffunb.2021.681631 (PMC10512348; doi:10.3389/ffunb.2021.681631)
Supplement: Supplementary Figure 13 — Growth analysis of A. niger reference strain (MA234.1), ΔtanR, ΔtanX, Δrce 17x, and ΔNRRL3_04659 on various carbon sources. Spores were point inoculated on minimal medium (MM) supplemented with the indicated carbon source (5 mM). Strains were grown for 5 days at 30°C before pictures were taken. [file Presentation_1.PPTX]

## Slide 1
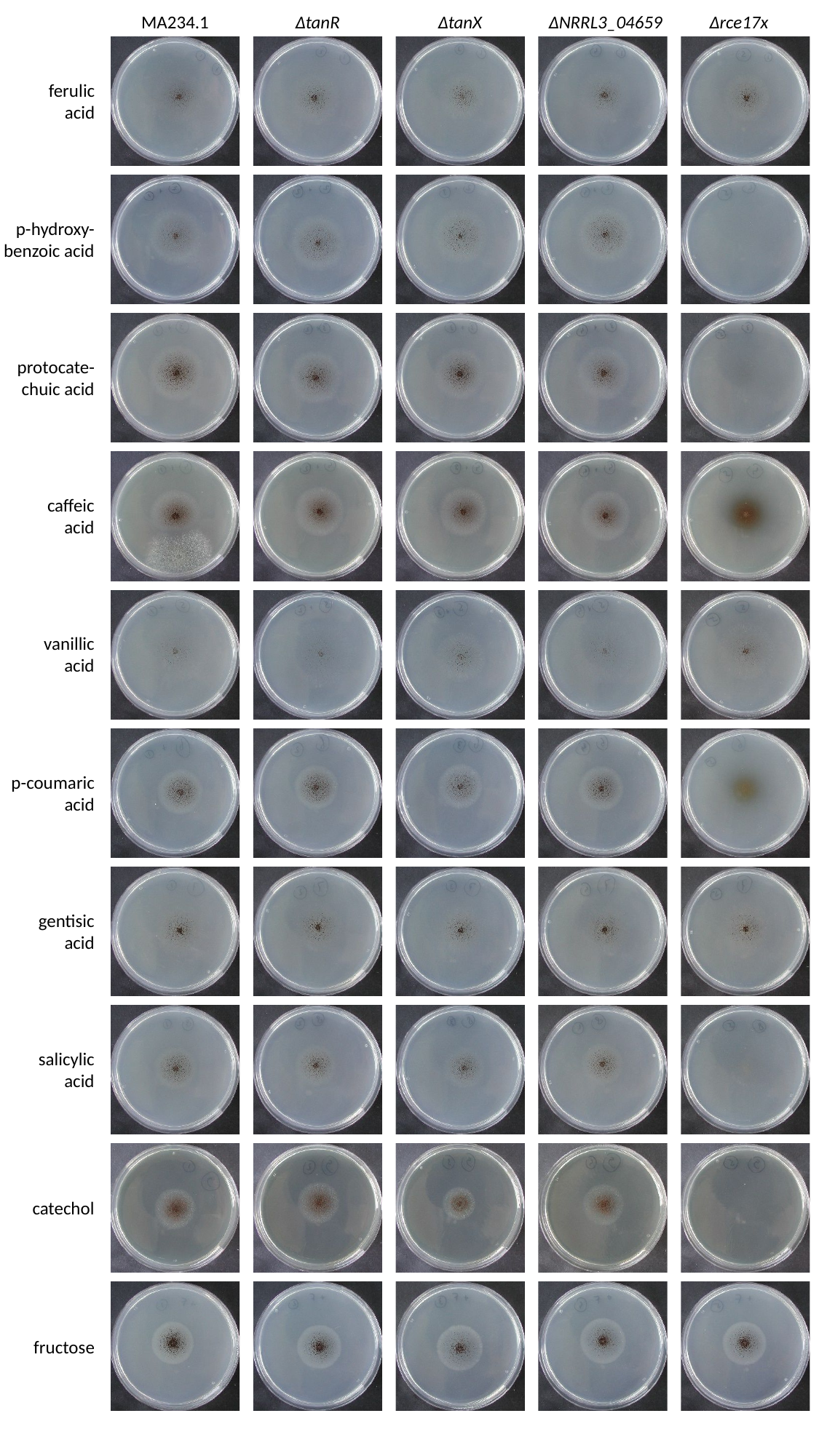

MA234.1
ΔtanR
ΔtanX
ΔNRRL3_04659
Δrce17x
ferulic
acid
p-hydroxy-
benzoic acid
protocate-
chuic acid
caffeic
acid
vanillic
acid
p-coumaric
acid
gentisic
acid
salicylic
acid
catechol
fructose
