# Supplementary figures and images for: Identification of a Conserved Transcriptional Activator-Repressor Module Controlling the Expression of Genes Involved in Tannic Acid Degradation and Gallic Acid Utilization in Aspergillus niger
Source: Front Fungal Biol. 2021 May 25;2:681631. doi: 10.3389/ffunb.2021.681631 (PMC10512348; doi:10.3389/ffunb.2021.681631)

## Slide 1
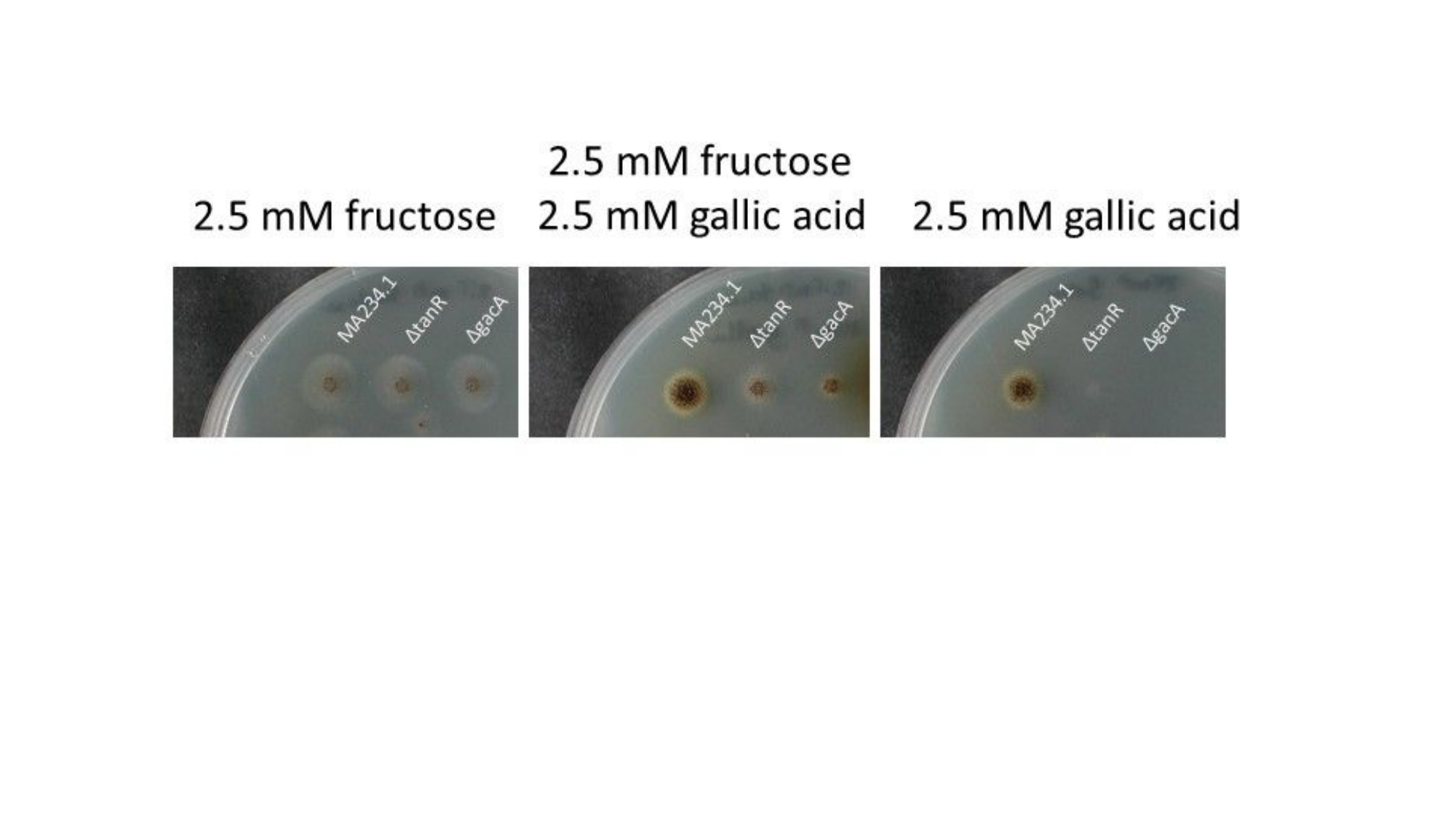

Supplement: Supplementary Figure 14 — Growth analysis of A. niger reference strain (MA234.1), ΔtanR, and ΔgacA strains on 2.5 mM fructose (left), 2.5 mM fructose and 25 mM gallic acid (middle), or 25 mM gallic acid (right). Spores were point inoculated on minimal medium (MM) supplemented with the indicated carbon source. Strains were grown for 3 days at 30°C before pictures were taken. [file Presentation_2.PPTX]
