# Supplementary material for: Identification of a Conserved Transcriptional Activator-Repressor Module Controlling the Expression of Genes Involved in Tannic Acid Degradation and Gallic Acid Utilization in Aspergillus niger
Source: Front Fungal Biol. 2021 May 25;2:681631. doi: 10.3389/ffunb.2021.681631 (PMC10512348; doi:10.3389/ffunb.2021.681631)
Supplement: Supplementary Table 3 — Gene clusters higher expressed in ΔtanX. [file Table_3.DOCX]

Supplemental Table 3: Gene clusters induced in *∆tanX* strain compared the parental strain (MA234.1)

| **Cluster 1** |  |  | TPM (MA234.1) | TPM *(∆tanX*) | FC DSeq2 | FDR |
| --- | --- | --- | --- | --- | --- | --- |
| NRRL3_01649 | An01g00800 | hypothetical protein | 0,00 | 0,03 | #N/A | n.a. |
| NRRL3_01650 | An01g00810 | hypothetical protein | 0,00 | 0,21 | #N/A | n.a. |
| NRRL3_01651 | An01g00820 | MFS-type sugar/inositol transporter | 0,05 | 0,41 | 3,28 | 5,10E-05 |
| NRRL3_01652 | An01g00850 | MFS-type sugar/inositol transporter | 115,21 | 802,40 | 7,44 | 1,81E-230 |
| NRRL3_01653 | An01g00860 | multicopper oxidase | 0,01 | 17,91 | 297,15 | 3,37E-150 |
| NRRL3_01654 | An01g00870 | prenyltransferase, UbiA family | 0,01 | 9,87 | 94,50 | 3,87E-77 |
| NRRL3_01655 | An01g00880 | O-methyltransferase, COMT-type | 0,00 | 620,98 | 5712,85 | 0,00E+00 |
| NRRL3_01656 | An01g00890 | hypothetical protein | 0,11 | 36,15 | 189,89 | 6,69E-167 |
| NRRL3_01657 | An01g00900 | Protein tyrosine phosphatase domain-containing protein | 4,12 | 4,98 | 1,32 | 7,13E-04 |
| NRRL3_01658 | An01g00910 | Nucleoside phosphorylase | 0,89 | 1,03 | #N/A | #N/A |
|  |  |  |  |  |  |  |
| **Cluster 2** |  |  |  |  |  |  |
| NRRL3_02175 | An01g06790 | hypothetical protein | 0,11 | 9,90 | #N/A | #N/A |
| NRRL3_02176 | An01g06800 | ceramidase | 69,22 | 45,49 | 0,73 | 3,81E-04 |
| NRRL3_02177 | An01g06810 | uncharacterized protein | 3,93 | 73,86 | 19,56 | 0,00E+00 |
| NRRL3_02178 | An01g06820 | bifunctional P-450:NADPH-P450 reductase | 0,20 | 7,78 | 14,44 | 2,60E-24 |
| NRRL3_02179 | An01g06830 | short-chain dehydrogenase/reductase | 0,35 | 5,57 | 8,80 | 2,20E-18 |
| NRRL3_02180 | An01g06840 | AMP-dependent synthetase/ligase | 0,09 | 8,41 | 27,96 | 2,97E-37 |
| NRRL3_02181 | An01g06850 | iron-containing alcohol dehydrogenase | 0,14 | 7,10 | 6,95 | 5,06E-13 |
| NRRL3_02182 | An01g06860 | phytanoyl-CoA dioxygenase | 0,08 | 11,60 | 2,70 | 4,02E-06 |
| NRRL3_02183 | An01g06870 | aminotransferase, class I/classII | 0,13 | 7,49 | 21,42 | 7,00E-33 |
| NRRL3_02184 | An01g06880 | NAD-dependent epimerase/dehydratase | 0,56 | 25,69 | 10,96 | 3,28E-18 |
| NRRL3_02185 | An01g06890 | non-ribosomal peptide synthetase-like protein | 0,28 | 16,30 | 11,93 | 1,96E-19 |
| NRRL3_02186 | An01g06900 | fungal-specific transcription factor | 4,59 | 12,63 | 2,80 | 7,60E-14 |
| NRRL3_02187 | An01g06910 | cytochrome P450 | 26,60 | 27,66 | 1,14 | 4,41E-02 |
| NRRL3_02188 | An01g06920 | ABC transporter | 132,80 | 146,06 | 1,21 | 5,28E-03 |
|  |  |  |  |  |  |  |
| **Cluster 3** |  |  |  |  |  |  |
| NRRL3_06145 | An02g01360 | mitochondrial inner membrane translocase subunit Tim17/Tim22/Tim23/peroxisomal protein PMP24 | 157,20 | 153,52 | #N/A | #N/A |
| NRRL3_06146 | An02g01350 | ankyrin repeat domain-containing protein | 0,44 | 0,63 | #N/A | #N/A |
| NRRL3_06147 | An02g01340 | hypothetical protein | 5,35 | 14,19 | 2,73 | 6,21E-14 |
| NRRL3_06148 | An02g01330 | NADH:flavin oxidoreductase/NADH oxidase family protein | 1,64 | 9,08 | 5,09 | 7,74E-23 |
| NRRL3_06149 | An02g01320 | hypothetical protein | 76,09 | 7053,53 | 93,15 | 0,00E+00 |
| NRRL3_06150 | An02g01300 | isochorismatase family protein | 0,50 | 58,41 | 40,73 | 1,26E-52 |
| NRRL3_06151 | An02g01290 | Ca2+/calmodulin-dependent/calcium-dependent protein kinase | 23,46 | 35,20 | 1,64 | 3,66E-17 |
| NRRL3_06152 | An02g01270 | MFS-type transporter | 34,64 | 29,20 | #N/A | #N/A |
|  |  |  |  |  |  |  |
| **Cluster 4** |  |  |  |  |  |  |
| NRRL3_08288 | An03g06670 | P-loop containing nucleoside triphosphate hydrolase | 46,17 | 71,70 | 1,60 | 2,60E-02 |
| NRRL3_08289 | An03g06660 | proton-dependent oligopeptide transporter | 1,00 | 1,67 | 1,75 | 8,36E-04 |
| NRRL3_08290 | An03g06650 | phospholipase C-like phosphodiesterase | 13,94 | 45,40 | 3,51 | 1,94E-75 |
| NRRL3_08291 | An03g06640 | hypothetical protein | 21,64 | 393,91 | 17,34 | 7,02E-130 |
| NRRL3_08292 | An03g06630 | carboxylesterase | 0,35 | 4,64 | 11,20 | 1,40E-42 |
| NRRL3_08293 | An03g06620 | flavin reductase-like domain-containing protein | 10,51 | 7,72 | 0,82 | 6,42E-02 |
| NRRL3_08294 | An03g06610 | hypothetical protein | 0,01 | 0,00 | #N/A | #N/A |
|  |  |  |  |  |  |  |
| **Cluster 5** |  |  |  |  |  |  |
|  |  |  |  |  |  |  |
| NRRL3_09052 | An12g01840 | p53-like transcription factor | 6,79 | 4,15 | 0,69 | 1,47E-02 |
| NRRL3_09053 | An12g01820 | ubiquitin carboxyl-terminal hydrolase | 0,86 | 1,31 | #N/A | #N/A |
| NRRL3_09054 | An12g01800 | glucan endo-1,3-alpha-glucosidase | 0,02 | 3,12 | 57,80 | 4,40E-64 |
| NRRL3_09055 | An12g01790 | hypothetical protein | 0,00 | 9,79 | 65,06 | 1,32E-58 |
| NRRL3_09056 | An12g01780 | F-box domain-containing protein | 0,81 | 7,31 | 8,24 | 1,70E-42 |
| NRRL3_09057 | An12g01770 | PAN-1 domain-containing protein | 0,00 | 27,98 | 79,82 | 3,94E-67 |
| NRRL3_09058 | An12g01760 | hypothetical protein | 0,00 | 0,00 | #N/A | #N/A |
| NRRL3_09059 | An12g01750 | uncharacterized protein | 0,00 | 0,00 | #N/A | #N/A |
|  |  |  |  |  |  |  |
| **Cluster 6** |  |  |  |  |  |  |
|  |  |  |  |  |  |  |
| NRRL3_10363 | An18g02590 | lipopolysaccharide kinase | 0,07 | 0,15 | #N/A | #N/A |
| NRRL3_10364 | An18g02600 | hypothetical protein | 12,02 | 3,75 | 0,37 | 5,69E-14 |
| NRRL3_10365 | An18g02610 | ankyrin repeat domain-containing protein | 0,00 | 0,02 | #N/A | #N/A |
| NRRL3_10366 | An18g02620 | hypothetical protein | 0,00 | 0,04 | #N/A | #N/A |
| NRRL3_10367 | An18g02630 | peptidase S8, subtilisin-related protein | 0,01 | 0,12 | #N/A | #N/A |
| NRRL3_10368 | An18g02650 | MFS-type transporter | 124,77 | 1120,01 | 6,50 | 4,67E-18 |
| NRRL3_10369 | 0 | methyltransferase, type 11 | 164,32 | 7146,12 | 10,84 | 4,37E-18 |
| NRRL3_10370 | 0 | fungal-specific transcription factor | 7,77 | 156,88 | 8,50 | 1,16E-15 |
| NRRL3_10371 | 0 | serine hydrolase FSH family protein | 57,68 | 3202,77 | 7,51 | 1,15E-13 |
| NRRL3_10372 | 0 | aldose 1-epimerase | 20,29 | 986,76 | 7,19 | 5,00E-13 |
| NRRL3_10373 | 0 | cytochrome P450 | 30,57 | 1836,48 | 9,23 | 4,66E-16 |
| NRRL3_10374 | 0 | zinc-type alcohol dehydrogenase | 62,20 | 3086,02 | 10,07 | 5,34E-17 |
| NRRL3_10375 | 0 | polyketide synthase, reducing | 16,25 | 887,55 | 9,89 | 8,03E-17 |
| NRRL3_10376 | An18g02680 | cytochrome P450 | 97,37 | 62,14 | 0,71 | 9,48E-05 |
| NRRL3_10377 | An18g02690 | multicopper oxidase | 7,66 | 3,11 | 0,54 | 9,75E-03 |
|  |  |  |  |  |  |  |
|  |  |  |  |  |  |  |
|  |  |  |  |  |  |  |
