# Supplementary material for: Identification of a Conserved Transcriptional Activator-Repressor Module Controlling the Expression of Genes Involved in Tannic Acid Degradation and Gallic Acid Utilization in Aspergillus niger
Source: Front Fungal Biol. 2021 May 25;2:681631. doi: 10.3389/ffunb.2021.681631 (PMC10512348; doi:10.3389/ffunb.2021.681631)
Supplement: Supplementary Table 4 — Expression values of genes encoding ring cleavages enzymes. [file Table_4.DOCX]

Supplemental Table 4. Expression values of putative ring opening enzymes

| NRRL3_ID | CBS513.88_ID | Description | TPM | TPM | FC | FDR |
| --- | --- | --- | --- | --- | --- | --- |
|  |  |  | WT average | *∆tanX* average | DSeq2  *∆tanX*/WT | Dseq2 |
| NRRL3_00984 | An14g04660 | extradiol ring-cleavage dioxygenase | 0,70 | 0,57 | n.a. | n.a. |
| NRRL3_03141 | An12g07530 | extradiol ring-cleavage dioxygenase | 2,75 | 3,35 | 1,32 | 7,31E-02 |
| NRRL3_04335 | An07g01390 | extradiol ring-cleavage dioxygenase | 0,42 | 0,20 | n.a. | n.a. |
| NRRL3_08632 | An03g02070 | extradiol ring-cleavage dioxygenase | 0,21 | 83,55 | 232,77 | 2,64E-204 |
| NRRL3_01405 | An13g02000 | intradiol ring-cleavage dioxygenase | 2,80 | 1,93 | n.a. | n.a. |
| NRRL3_02644 | An01g12310 | intradiol ring-cleavage dioxygenase | 11,04 | 27,30 | 2,61 | 2,08E-19 |
| NRRL3_04787 | An07g07280 | intradiol ring-cleavage dioxygenase | 16,89 | 14,22 | n.a. | n.a. |
| NRRL3_05330 | An02g11530 | intradiol ring-cleavage dioxygenase | 1,76 | 1,56 | n.a. | n.a. |
| NRRL3_00414 | An09g05150 | Intradiol ring-cleavage dioxygenase core domain-containing protein | 0,45 | 0,16 | 0,58 | 9,28E-02 |
| NRRL3_02896 | An12g10740 | Intradiol ring-cleavage dioxygenase core domain-containing protein | 0,17 | 0,08 | n.a. | n.a. |
| NRRL3_04277 | An07g00700 | Intradiol ring-cleavage dioxygenase core domain-containing protein | 0,52 | 25,82 | 38,30 | 3,18E-111 |
| NRRL3_02496 | An01g10640 | dioxygenase | 2,28 | 1,64 | n.a. | n.a. |
| NRRL3_02522 | An01g10910 | dioxygenase | 0,01 | 0,07 | n.a. | n.a. |
| NRRL3_03148 | An12g07430 | dioxygenase | 0,68 | 0,26 | 0,55 | 3,14E-02 |
| NRRL3_03326 | An12g05150 | dioxygenase | 0,82 | 0,80 | n.a. | n.a. |
| NRRL3_05564 | An02g08560 | dioxygenase | 0,66 | 0,33 | n.a. | n.a. |
| NRRL3_11743 | An06g00210 | dioxygenase | 0,40 | 0,21 | n.a. | n.a. |
